# Integrated stress response plasticity governs normal cell adaptation to chronic stress via the PP2A-TFE3-ATF4 pathway

**DOI:** 10.1038/s41418-024-01378-3

**Published:** 2024-09-30

**Authors:** Rita A. Avelar, Riya Gupta, Grace Carvette, Felipe da Veiga Leprevost, Medhasri Jasti, Jose Colina, Jessica Teitel, Alexey I. Nesvizhskii, Caitlin M. O’Connor, Maria Hatzoglou, Shirish Shenolikar, Peter Arvan, Goutham Narla, Analisa DiFeo

**Affiliations:** 1 Department of Pathology, The University of Michigan, Ann Arbor, MI USA; 2 Rogel Cancer Center, The University of Michigan, Ann Arbor, MI USA; 3 Department of Computational Medicine and Bioinformatics, University of Michigan, Ann Arbor, MI USA; 4 Department of Internal Medicine, Division of Genetic Medicine, University of Michigan, Ann Arbor, MI USA; 5 Department of Genetics and Genome Sciences, Case Western Reserve University, Cleveland, OH USA; 6 Duke-NUS Medical School, Singapore, Singapore; 7 Duke University School of Medicine, Durham, NC USA; 8 Division of Metabolism Endocrinology and Diabetes, University of Michigan Medical Center, Ann Arbor, MI USA; 9 Department of Obstetrics and Gynecology, University of Michigan, Ann Arbor, MI USA

**Keywords:** Cell biology, Protein folding, Tumour-suppressor proteins, Drug development, Translational research

## Abstract

The integrated stress response (ISR) regulates cell fate during conditions of stress by leveraging the cell’s capacity to endure sustainable and efficient adaptive stress responses. Protein phosphatase 2A (PP2A) activity modulation has been shown to be successful in achieving both therapeutic efficacy and safety across various cancer models. However, the molecular mechanisms driving its selective antitumor effects remain unclear. Here, we show for the first time that ISR plasticity relies on PP2A activation to regulate drug response and dictate cellular survival under conditions of chronic stress. We demonstrate that genetic and chemical modulation of the PP2A leads to chronic proteolytic stress and triggers an ISR to dictate whether the cell lives or dies. More specifically, we uncovered that the PP2A-TFE3-ATF4 pathway governs ISR cell plasticity during endoplasmic reticular and cellular stress independent of the unfolded protein response. We further show that normal cells reprogram their genetic signatures to undergo ISR-mediated adaptation and homeostatic recovery thereby avoiding toxicity following PP2A-mediated stress. Conversely, oncogenic specific cytotoxicity induced by chemical modulation of PP2A is achieved by activating chronic and irreversible ISR in cancer cells. Our findings propose that a differential response to chemical modulation of PP2A is determined by intrinsic ISR plasticity, providing a novel biological vulnerability to selectively induce cancer cell death and improve targeted therapeutic efficacy.

## Introduction

Eukaryotic cells can adapt to various environmental challenges promptly and efficiently to promote cell survival. However, under chronic stress conditions, cells that cannot activate a sufficient adaptive response to mitigate stress and restore homeostasis are targeted for programmed cell death. One of the predominant signaling pathways known to regulate such adaptive response is the known as the integrated stress response (ISR), a sensor mechanism that promptly reacts to extrinsic and intrinsic stress factors including [1] oxygen and amino acid deprivation [2] viral infection [3] glucose imbalances [4] oncogenic activation, and [5] overload/ accumulation of misfolded proteins in the endoplasmic reticulum (ER) thereby triggering an unfolded protein response (UPR) [1–7]. When the ISR is unable to sustain stress adaptation mechanisms, anti-survival components are activated to execute cell death. ATF4 is the master regulator of ISR-mediated cellular adaptation during stress conditions, dictating the transcriptional activation of several genes involved in homeostatic recovery. A stress response, amino acid biosynthesis, and CHOP-mediated autophagy and apoptotic signatures follow, ultimately dictating cell survival [5, 8**–**10]. This decision is dictated by the nature, duration, space, and magnitude of both the stress and the induced ISR. Thus, ISR reversibility is an essential component of cell survival outcomes and decision-making, being instrumental for cell survival [11]. At basal states, cancer cells develop the unique ability to favor pro-survival phenotypes through the exploitation of ISR signaling, allowing them to withstand the high levels of protein synthesis driven by exacerbated oncogenic demand [12, 13]. Aberrant responses to activated ISR signaling have been widely implicated in cellular transformation, cancer development, metastasis, and chemoresistance mechanisms in many human malignancies. In contrast, normal cells maintain greater basal adaptive plasticity, which enables them to recover from chronically induced ISR and restore their identity and primary function [14].

Protein phosphatase 2A is a heterotrimeric serine/threonine tumor suppressive protein phosphatase that is comprised of three main components: PP2A-A—the scaffolding subunit, providing a nucleating platform through its conformational flexibility for heterotrimer formation—, PP2A-C—the catalytic subunit, which provides enzymatic activity directed against phosphorylated serine and threonine residues—, and PP2A-B—one of fifteen structurally distinct regulatory subunits that directs substrate specificity [15–17]. Given that cellular homeostasis is dysregulated in human cancers due to imbalances in protein phosphorylation, which results in global cellular signaling perturbations, the therapeutic targeting of phosphatases such as PP2A has emerged as an effective therapeutic strategy to reestablish cellular homeostasis [18–28]. DT-061 is a PP2A modulator that regulates this tumor suppressor’s activity by selectively binding to a unique pocket at the interface of the PP2A-Aα, Cα, and B56α subunits [29]. DT-061 demonstrates potent anticancer properties in numerous cancer models with high tolerability profiles, even in long-term in vivo dosing studies [21–28]. However, the role of DT-061 and other PP2A modulators in regulating human stress-mediated responses has yet to be elucidated, remaining unclear whether this could explain the promising safety profiles previously observed across numerous cancer models.

Despite the evident efficacy in targeted therapeutic strategies, a significant challenge resides in ensuring selective and specific oncogenic toxicity while sparing healthy tissues. The present study has sought to elucidate the mechanisms through which PP2A modulation specifically induces cell death in cancer cells. Herein, we demonstrated that DT-061 triggers ISR to dictate cell death. Accordingly, under reversible and chronic stress conditions induced with DT-061 treatment, non-malignant human cells undergo transcriptional and translational reprogramming, leveraging their inherent ISR plasticity to survive and restore homeostasis, a mechanism previously observed to occur in normal tissues to overcome cellular stress. This reprograming represses stress-mediated cell death, serving as an adaptive homeostatic mechanism that enables non-malignant tissues to restore basal functions, reinstate their cellular identity, and ultimately survive. Conversely, global proteomics and RNA-seq analyses revealed that PP2A modulation via DT-061 induces irreversible ISR in cancer cells, preventing them to resort to adaptive mechanisms that would enable cell survival. Therefore, whereas DT-061 elicits ISR downstream pathways associated with chronic stress and cell death responses in cancer cells, it simultaneously activates distinct ISR-mediated signatures that confer adaptive and protective mechanisms in non-transformed cells, contributing to their survival. Mechanistically, we uncovered that DT-061-mediated modulation of PP2A induced the ISR through the dephosphorylation of TFE3, resulting in chronic upregulation of ATF4 and CHOP to inhibit stress recovery and induce cell death in cancer cells. Importantly, our current studies provide evidence that the PP2A-TFE3-ATF4 axis is a molecular process essential to dictate ISR plasticity, supporting a promising avenue for cancer-selective therapies based on manipulating PP2A-mediated irreversible ISR, while also providing novel insight into DT-061’s antitumor properties.

## Results

### Stress response and cell death pathways are enriched in cancer cells treated with DT-061

Our group recently discovered that over 90% of high-grade serous cancers (HGSC) harbor loss of heterozygosity in PP2A genes, rendering them highly sensitive to the PP2A modulator DT-061, both as a monotherapy and in combination with PARP inhibitors [21]. We established that DT-061 stabilizes the remnant copy of PP2A-Aα, thus biasing specific PP2A heterotrimeric pools towards its tumor suppressive function. Despite the potent oncogenic cytotoxicity induced by DT-061, normal cells were able to circumvent pro-death signals, enhancing the therapeutic index window and tolerability profiles in vivo [21–28]. In this study, our aim is to explore the molecular mechanism by which this class of drugs are selectively lethal to cancer cells while sparing normal tissues.

**Fig. 1:**
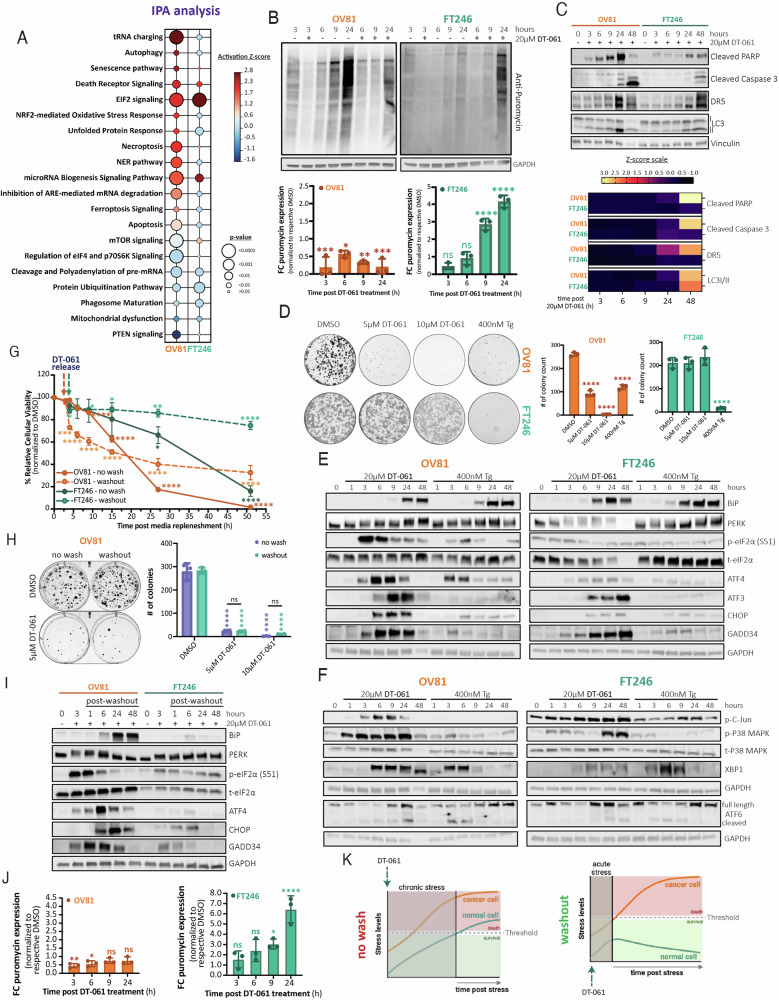
DT-061-mediated activation of the ISR pathway dictates cellular survival by specifically inducing irreversible cell death in cancer cells. **A** Ingenuity Pathway Analysis (IPA) identifies ISR and ER stress-related and cellular adaptation pathways such as t-RNA charging, EIF2, NRF2-mediated oxidative stress response, and UPR, as well as cell death including autophagy, senescence, death receptor, and necroptosis signaling, cascades among the topmost significantly induced pathways in HGSC cancer cells after 3 h of DT-061 relative to DMSO. The color scale represents Z-score activation and circles represent the statistical significance. **B** Puromycin 10-min pulse with 50-min chase labeling experiments in the presence of DMSO or DT-061 after 3, 6, 9, and 24 h incubation, to measure newly translated protein. Western blotting analysis was used to quantify puromycin incorporation and GAPDH as the loading control (top). Whole lane quantification was graphed as fold change in puromycin expression relative to respective DMSO over time (bottom). Data presented as the mean ± SD (*n* = 3), (one-way ANOVA with multiple comparisons, comparing the mean of each column with the mean of 3 h DMSO control column (not represented) for respective cell line, ns > 0.05, *p < 0.05, **p < 0.01, ***p < 0.001, ****p < 0.0001). **C** Western blotting analysis (top) evaluating the expression of cell death, apoptosis, and autophagy markers (cleaved PARP, cleaved Caspase 3 and DR5, and LC3I/II, respectively) upon DT-061 treatment. Representation by heatmap of Z-score values (bottom) obtained after western blot quantification using image J and normalization to Vinculin as the loading control. **D** Representative images of the clonogenic assay testing OV81 and FT246 cells’ ability to form colonies in the presence of DMSO, 5 µM of DT-061, 10 µM of DT-061, or 400 nM of Tg for two weeks (left). Quantification is represented (right) and calculated as the mean ± SD (*n* = 3), (one-way ANOVA with multiple comparisons, comparing the mean of each column with the mean of DMSO control for its respective cell line, ****p < 0.0001). **E**, **F** Western blotting analysis assessing DT-061-mediated activation of every arm of the UPR in comparison to Thapsigargine (Tg) (positive control). **E** PERK, and **F** IRE1α and ATF6 pathways and respective downstream targets were thoroughly analyzed under different exposure times to DT-061 or Tg in cancer (OV81) and non-malignant (FT246) cellular models. **G** OV81 and FT246 cells were pre-incubated with DMSO or 20 µM of DT-061 for 3 h, followed by continuous treatment (no-wash) or replenished with fresh media lacking drug (washout). Cellular viability was measured over time using cell titer glo and the cell viability percentage was calculated relative to 0 h. Data presented as the mean ± SD (*n* = 3) (one-way ANOVA with multiple comparisons, comparing the mean of each data point with the mean of 0 h time point (100%) for its respective cell line, *p < 0.05, **p < 0.01, ****p < 0.0001). **H** OV81 clonogenic assays evaluating the effect of DMSO versus 5 µM and 10 µM of DT-061 treatment in no-wash and washout conditions. No-wash conditions were replenished with fresh drug media every 3 days whereas washout cells were treated with drug once for 3 consecutive days followed by fresh drug-free media every three days until the end of the experiment (top). Quantification is represented (bottom) and calculated as the mean ± SD (*n* = 3), (one-way ANOVA with multiple comparisons, comparing the mean of each column with the mean of their own respective DMSO control column, ****p < 0.0001). **I** Western blotting analysis evaluating the molecular mechanism and profiles of DT-061 post washout versus no-wash downstream of the ISR and PERK pathway. **J** Graphs representing the fold change in puromycin signal relative to DMSO for OV81 (top) and FT246 (bottom), with 10-min pulse and 50-min chase puromycin labeling for the washout protein lysates collected under the same conditions as I). Data presented as the mean ± SD (*n* = 3), (one-way ANOVA with multiple comparisons, comparing the mean of each column with the mean of 3 h DMSO control column (not represented) for its own respective cell line, ns > 0.05, *p < 0.05, **p < 0.01, ****p < 0.0001). **K** Schematic demonstrating the differences in cancer and normal cells responses to chronic (left) versus acute (right) stress mediated by DT-061. If stress is chronically induced by constant exposure to DT-061, both malignant and non-malignant cells will be targeted for cell death. However, as cancer cells have a higher yield of baseline protein expression to sustain oncogenic signaling, thus leading to lower thresholds to homeostatic imbalances and inherently higher baseline stress levels, DT-061-mediated acute stress will still lead to death. Oppositely, normal cells are capable of rapidly adapting and promptly responding to DT-061-mediated ER stress cues under acute conditions, inducing pro-survival signals that eventually restore homeostasis. Schematic designed on Biorender.

Ingenuity Pathway Analysis (IPA) of global proteomics studies revealed that DT-061 treatment significantly induced cellular stress responses and death signaling pathways in patient-derived HGSC cells (OV81) compared to non-malignant fallopian tube cells (FT246) (Fig. 1A). More specifically, the Death Receptor and EIF2 signaling pathways were activated in both malignant and non-cancerous contexts, whereas t-RNA charging, NRF2-mediated oxidative stress, and the unfolded protein response (UPR) were exclusively upregulated in cancer cells following DT-061 treatment. Furthermore, only cancer cells exhibited increased apoptosis and SUMOylation after treatment with DT-061 (Supplementary Fig. 1A–C). In contrast, translation, RNA processing, and protein transport were uniquely and significantly enriched in non-malignant cells, suggesting distinct downstream signatures that regulate cell survival are triggered with DT-061 contingent upon their malignant context. These results were also observed in the RNA-seq analysis of a non-small cell lung cancer (NSCLC) cell line, H358 [30]. Herein, we found that DT-061 treatment in NSCLC cells significantly upregulated genes associated with endoplasmic reticulum (ER) stress, UPR, and integrated stress response (ISR), including HERPUD1, ATF3, DDIT3 (CHOP), TRIB3, PPP1R15A (GADD34), IRE1α, ATF6, EIF2, PERK, and ATF4 activated genes (Supplementary Fig. 1D–F). To validate the functional impact of these distinct signatures, we evaluated DT-061’s impact on the ER-to-Golgi transport using the Gaussia luciferase secretory assay [31]. We found that DT-061 disrupted luciferase protein secretion as effectively as the ER-to-Golgi transport inhibitor brefeldin A (BFA) (Supplementary Fig. 1G, H). Interestingly, we observed that DT-061 increased Gaussia mRNA levels in both lines but decreased protein expression only in cancer cells, suggesting that the lack of protein secretion in these cells may be due to translation inabilities (Supplementary Fig. 1I–K). To validate these results, we next assessed the impact of DT-016 on overall translation using a pulse-chase puromycin labeling SUrface SEnsing of Translation (SUnSET) assay, that measures nascent polypeptide chain formation (Fig. 1B). Consistently, this assay showed that cancer cells treated with DT-061 displayed a marked and irreversible attenuation in protein translation whereas normal cells induced an effective translational recovery.

Collectively, these results suggest that normal and cancer cells display a differential response to DT-061. While normal cells adapt to survive, cancer cells demonstrate compromised translation capabilities, subsequently activating pro-apoptotic stress responses due to their inability to initiate parallel adaptive mechanisms, ultimately leading to cell death.

### DT-061 activates ISR signaling in HGSC and FT cells to dictate cell survival

The ER stress response involves a complex interconnected network that regulates cellular survival outcomes by predominantly activating PERK, one of four kinase regulators of the ISR. Under chronic stress, these signaling pathways lead to the accumulation of CHOP, a well-established ISR transcriptional activator of cell death (Supplementary Fig. 2A). Given the observed effects of DT-061 on the ISR, we then examined the three major cell death mechanisms activated by this pathway, including apoptosis (cleaved PARP, cleaved Caspase 3), stress-mediated death (DR5), autophagy (LC3I/II), and reactive oxygen species (ROS) accumulation signals. We found that DT-061 significantly induced all death-associated pathways more rapidly and with greater intensity in cancer compared to normal cells (Fig. 1C and Supplementary Fig. 2B). Annexin V-PI staining, autophagosome maturation, and ROS assays further corroborated these results (Supplementary Fig. 2C–F). We next compared the effects of DT-061 to those of thapsigargin (Tg), a potent ER stress/UPR inducer via the inhibition of the sarco/endoplasmic Ca^2+^ ATPase (SERCA) pump [32]. Notably, Tg triggered similar robust cytotoxic effects in both normal and cancer cells, unlike DT-016 which significantly decreased colony formation in cancer cells but remained non-toxic to the non-malignant counterparts (Fig. 1D and Supplementary Fig. 2G, H*)*.

To investigate the impact of each arm of the UPR in driving the observed cancer-specific effects of DT-061, we examined protein expression patterns of PERK, IRE1α, ATF6, and their downstream targets. As expected, Tg triggered all three pathways in both cancer and normal cells, which was eventually restored to baseline levels upon feedback activation *(*Fig. 1E, F, Supplementary Fig. 2I–K). Interestingly, DT-061 treatment led to PERK hyperphosphorylation as early as 1 h, subsequently triggering eIF2α phosphorylation (Serine-51) and ATF4, ATF3, CHOP, BiP, and GADD34 protein expression. Interestingly, induction of the PERK and IRE1α pathways in DT-061-treated FT cells had a comparative and significantly attenuated response.

### Chronic stress induced by DT-061 is irreversible and determines cancer cells’ survival outcomes

Thus far, our data suggests that ISR is a crucial component of the cellular response to stress induced by DT-061, predominantly in malignant contexts. Particularly, cancer cells exhibit a limited capacity in triggering adaptive mechanisms to restore cellular homeostasis, resulting in pro-death signals. In contrast, non-transformed cells engage adaptive homeostatic mechanisms, having the inherent ability to recover from stressful occurrences by selectively activating specific feedback mechanisms that regulate cellular survival. To further investigate the adaptive mechanisms driving the differential response between cancer and normal cells, we performed washout studies. We observed that continuous (no wash) DT-061 treatment in both cancer and non-malignant cell led to cell death by 48 h (100% and 85%, respectively) (Fig. 1G). However, cancer cells were unable to recover after washout conditions, unlike normal cells, which showed a ~60% recovery in viability after the drug was released (Fig. 1G, H*)*. To elucidate which molecular signatures explained these phenotypic differences, we evaluated protein and RNA expression changes in the ISR pathway and death markers for both washout and no wash conditions (Fig. 1I, Supplementary Fig. 2L–N). Strikingly, despite the PERK phosphorylation attenuation at 24 h post washout, all targets canonically downstream of eIF2α phosphorylation including ATF4, CHOP, and GADD34 remained transcriptionally and translationally activated specifically in cancer cells. The sustained suppression of these protein targets following drug washout suggests that cancer cells are inherently incapable of promptly restoring homeostasis, thus leading to irreversible cell death, despite DT-061 removal (Supplementary Fig. 2L). In contrast, FT246 cells showed reversibility of stress by significantly recovering ATF4, CHOP, GADD34, and BiP expression. Interestingly, in cancer cells, ATF4 and CHOP mRNA expression were significantly induced after DT-061 treatment and only fully rescued to baseline levels after 24 h of drug washout (Supplementary Fig. 2M—left panel). Conversely, in normal cells, while ATF4 eventually returns to baseline, CHOP levels remain elevated and are not restored even after 24 h post DT-061 washout (Supplementary Fig. 2M— right panel). Consistent with our no-washout results, nascent protein translation after drug washout revealed that normal cells still sustain stress-induced translation levels, while translation in OV81 cells remains inhibited (Fig. 1J). Despite elevated CHOP and ATF4 mRNA, FT246’s translational machinery selectively limits ISR gene production by enhancing overall protein translation, a mechanism not observed in cancer cells. Most importantly, these molecular phenotypes extend to other ovarian cancer and non-malignant models (Supplementary Fig. 2O, P). Thus, our data suggest that prolonged exposure to DT-061 can result in both malignant and non-transformed cells sharing the same outcome: cell death (Fig. 1K—left panel). However, normal cells can quickly adapt to acute stressors and dynamic environments, triggering pro-survival signals that facilitate the activation of adaptive rheostatic mechanisms owing to their increased basal ISR plasticity (Fig. 1K—right panel).

### PP2A-mediated regulation of the ATF4-CHOP pathway via DT-061 is independent of the UPR

**Fig. 2:**
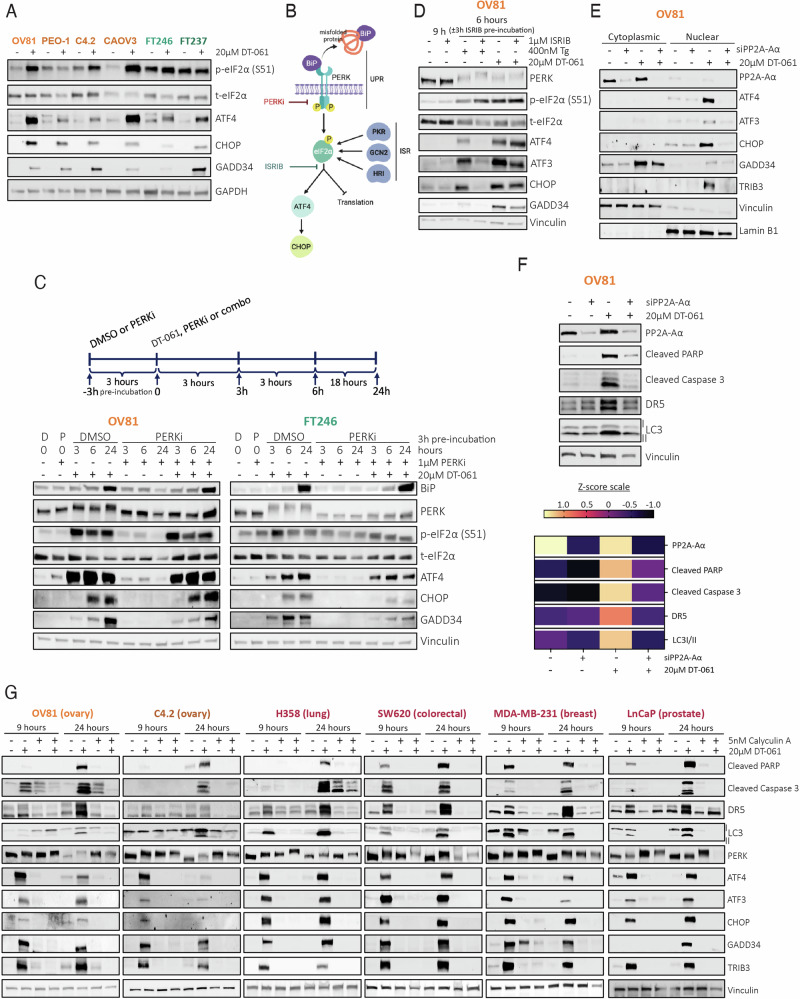
Chronic DT-061-Induced ATF4-CHOP activation triggers cancer cell death independently of PERK activity and eIF2α phosphorylation. **A** Western blotting analysis evaluating the effect of DT-061 treatment on eIF2α phosphorylation, ATF4, and CHOP expression in multiple HGSC and FT lines to evaluate dependency profiles. OV81, PEO-1, PEO-C4.2, and CAOV3 represent HGSC models (shades of orange) while FT246 and FT237 represent non-malignant fallopian tube tissues (shades of green). **B** Schematic representing the two well-established canonical pathways that phosphorylate eIF2α resulting in ATF4 and CHOP increased expression: PERK (via the UPR) and PKR, GCN2, and HR (as part of the Integrated Stress Response (ISR)). Chemical inhibitors utilized to test PERK and eIF2α contribution to DT-061-mediated ATF4 and CHOP activation are also represented. **C** Western blot analysis assessing the role of PERK activation and subsequent UPR regulation in OV81 and FT246 cells in the presence of DT-061. Cells were pre-incubated with either DMSO or 1 µM of PERK inhibitor (PERKi) for 3 h. Subsequently, DT-061 (DMSO pre-incubated), fresh PERKi, or the combination of the two drugs (PERKi pre-incubated) were added and cells were harvested after 3, 6, and 24 h of the second round of drug exposure. PERK pathway activity was measured by assessing its hyperphosphorylation state (band shift) and downstream targets’ expression. **D** Western blot analysis assessing the role of p-eIF2α downstream signaling and its impact in the activation of ATF4 and CHOP proteins in OV81 cells after treatment with DT-061. Cells were pre-incubated with either DMSO or 1 µM of ISR Inhibitor (ISRIB) for 3 h. Cells were then treated with either Tg or DT-061 after both DMSO and ISRIB pre-incubation and harvested at 6 h post second treatment round (total of 9 h). Expression of ATF4 and its downstream targets were evaluated under each of these treatment conditions and quantified (in Supplementary Fig. 2R). **E** A pool of siRNAs was used to knockdown PP2A-Aα protein expression for 24 h. Western blotting analysis was used to assess cytoplasmic versus nuclear localization as well as expression of the ATF4 and its downstream target genes after DT-061 treatment in both siControl (negative control) and siPP2A-Aα. **F** Whole cell lysate of the conditions previously described in (**E**) was also collected to evaluate the expression of cell death markers and ER-associated autophagy. Western blotting analysis was performed (top) and represented as z-score values in a heatmap obtained through western blot quantification (bottom). Calculated z-scores were obtained from protein quantification of three independent biological replicates. **G** PP2A chemical inhibitor calyculin A, was used to evaluate DT-061’s anticancer effects and their dependency on PP2A in other HGSC, lung, prostate, breast, and colorectal lines. Cells were pre-incubated with either DMSO or 5 nM of calyculin A for 1 h.

Our next step was to investigate how DT-061 could cause cancer cells to chronically activate ATF4 and CHOP, even after drug washout. Although all HGSC and FT lines tested showed ATF4 and CHOP protein upregulation with DT-061 treatment (Fig. 2A), p-eIF2α expression was not consistent across all different ovarian lines. Thus, we wanted to evaluate whether the activation of these gene products where independent of PERK or other genes that regulate p-eIF2α levels. Using a PERK inhibitor (PERKi, Fig. 2B) prior to DT-061 exposure, we found that PERK activation was effectively repressed (as observed by the reduced gel shift, i.e., suppressed hyperphosphorylation). However, phosphorylation of eIF2α persisted, which was accompanied by increased expression of ATF4, CHOP, and GADD34, to the same extent as DT-061 alone (Fig. 2C). This suggests that ISR activation mediated by DT-061 is independent of PERK activity. Moreover, PERKi co-treatment with DT-061 yields similar effects on cellular viability as DT-061 alone, showing that PERK inhibition is unable to prevent a stress response in cancer cells (Supplementary Fig. 2Q—left panel). Conversely, as non-malignant cells have an increased basal state of stress response due to higher inherent levels of p-eIF2α expression, FT246 cells have reduced viability over time with PERKi alone or in combination with DT-061 (Supplementary Fig. 2Q—right panel).

To then test whether p-eIF2α activity is required for the DT-061-mediated ATF4, CHOP, and cell death, we used the general ISR Inhibitor (ISRIB), a molecule that inhibits the bioactivity of p-eIF2α to suppress ISR signaling [33]. As expected, we found that ISRIB pre-incubation rescued the expression of ATF4, ATF3, CHOP, GADD34, and TRIB3 proteins induced by Tg alone [34] (Fig. 2D and Supplementary Fig. 2R). However, it failed to block the induction of ATF4, ATF3, CHOP, GADD34, and TRIB3 proteins induced by DT-061 treatment in cancer cells. These findings imply that DT-061 triggers the ATF4-CHOP pathway independently of PERK and eIF2α phosphorylation, thus involving an alternative mechanism that regulates the observed ISR molecular signatures. Given this outcome, we then sought to confirm that DT-061-mediated induction of ATF4 was modulated by PP2A activity. We established that PP2A inhibition by siRNA-mediated knockdown of PP2A-Aα, an essential component of PP2A holoenzyme activity, significantly abrogated the ability of DT-061 to activate or translocate these ER targets into the nucleus (Fig. 2E). In addition, cell death and autophagy markers were also found to be significantly rescued upon PP2A-Aα knockdown after DT-061 treatment (Fig. 2F). Remarkably, these effects were further confirmed in 4 additional cancer types (lung, colorectal, breast, and prostate) corroborating that DT-061’s oncogenic specific cytotoxic effects are universal to various malignant contexts (Fig. 2G).

### PP2A-dependent TFE3 activation of ATF4 and CHOP expression regulates cell survival

**Fig. 3:**
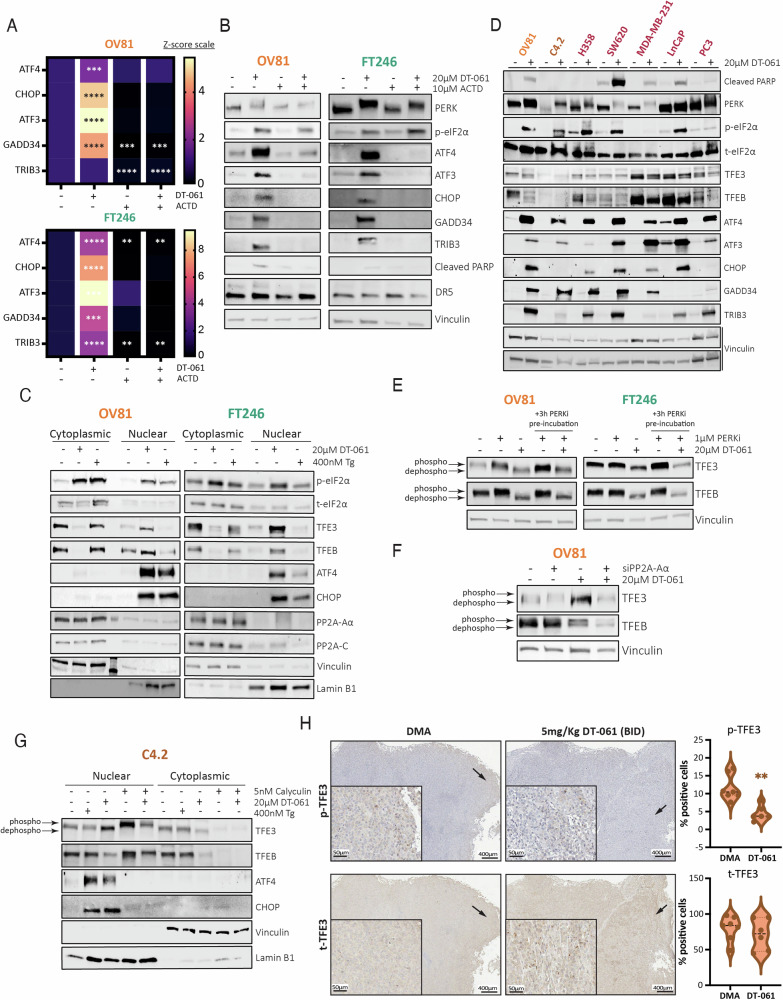
PP2A-dependent activation of ATF4 and CHOP expression is transcriptionally regulated by TFE3. **A** mRNA analysis of OV81 and FT246 cell lines assessing transcriptional expression and repression of ATF4, CHOP, ATF3, GADD34, and TRIB3 genes after DT-061 treatment with or without actinomycin D (ActD) pre-incubation, a potent transcription inhibitor that intercalates with the DNA to prevent RNA Polymerase binding. **B** Western blotting analysis evaluating the protein expression dependency on the transcriptional regulation induced by DT-061 in the OV81 cell line. **C** Western blotting analysis evaluating cytoplasmic versus nuclear localization and protein expression of TFEB/3, ATF4, and its downstream target genes after 6 h of DT-061 or Tg treatment in OV81. **D** Western blotting analysis evaluating the effects of DT-061 on the protein expression of TFE3, TFEB, and ISR downstream targets in lung (H358), colorectal (SW620), breast (MDA-MB-231), and prostate (LnCaP and PC3) cell lines. **E** Protein lysates from experiments represented in Fig. 2C were reanalyzed to evaluate TFEB/3 dephosphorylation status and overall expression under PERKi, DT-061, and combination conditions. Western blotting analysis helped evaluate TFEB/3’s dependency on the UPR-dependent regulation via Calcineurin, in the presence of DT-061. **F** Protein lysates from Fig. 2F evaluating TFEB/3 expression and dephosphorylation status upon DT-061 treatment, and thus evaluate dependency profiles of DT-061-mediated TFEB/3 effects on PP2A, the UPR-independent mechanism. **G** Western blotting analysis in C4.2 HGSC cell line evaluating nuclear versus cytoplasmic localization and protein expression of TFEB/3, ATF4, and CHOP after 6 h of Tg, DT-061, calyculin A, or DT + calyculin A treatments. **H** Representative histology pictures with immunohistochemistry staining (brown) of phospho and total TFE3 for DMA or DT-061 treated tumors from previously published OV81 PDX efficacy studies [21].

Given that DT-061-mediated activation of the ATF4-CHOP pathway does not fully rely on eIF2α phosphorylation, although is highly dependent on PP2A activity to trigger cancer cell death, we next ascertained whether these effects were mediated at the transcript level or via alternative mechanisms. Using actinomycin D (ActD), a potent transcription inhibitor, we found that in both cancer and normal cell lines, ActD inhibits DT-061-stimulated mRNA levels of ATF4 and all its downstream targets (Fig. 3A). Interestingly, co-treatment of ActD with DT-061 also restored the increased expression of ATF4, ATF3, CHOP, GADD34, and TRIB3 proteins back to baseline levels, while PERK and p-eIF2α post-translational regulation were unaffected (Fig. 3B). ATF4 protein half-life remained unchanged in both cancer and normal cells after DT-061 or Tg exposure (Supplementary Fig. 3A–C). Interestingly, however, DT-061 caused a significant increase in CHOP protein half-life specifically in cancer cells, which was not observed with Tg treatment (Supplementary Fig. 3D, F). Conversely, in normal cells, CHOP’s half-life increased with both DT-061 and Tg treatment, suggesting that, in addition to the considerable effect of DT-061 on mRNA targets of the ATF4 signaling pathway, there may be subtle additional mechanisms that impact on CHOP protein levels.

To understand the molecular mechanism underlying ATF4 transcriptional upregulation upon DT-061 treatment, we investigated the role of Transcription Factor E3 (TFE3) and EB (TFEB), recently recognized as novel crucial components of the ISR pathway and direct regulators of ATF4 and CHOP transcriptional regulation [35]. Interestingly, it has been suggested that PP2A [36] and calcineurin [35, 37] can regulate the shuttling of these proteins from the cytoplasm into the nucleus via dephosphorylation to transcriptionally activate their downstream targets (Supplementary Fig. 3F). We found that cellular TFE3 and TFEB were robustly dephosphorylated and translocated into the nucleus specifically upon DT-061 treatment and not with Tg (Fig. 3C). These findings were replicated in several additional cancer lines, providing further evidence that this mechanism is relevant in multiple different cancer types and not exclusive to HGSC (Fig. 3D). Next, we assessed whether DT-061-triggered effects on TFE3 and TFEB were specifically mediated by PP2A modulation and found that PERK inhibition had no effect on DT-061 mediated dephosphorylation of TFE3 and TFEB (Fig. 3E). However, PP2A-Aα knockdown was sufficient to block DT-061 induced dephosphorylation of TFE3 but not dephosphorylated TFEB (Fig. 3F), highlighting that the DT-061’s effects are specifically dependent on TFE3, and its activation relies on PP2A activity. Interestingly, using an additional ovarian cancer cell line we observed that inhibition of PP2A using calyculin A resulted in a significant upregulation of phosphorylated TFE3 with unique nuclear retention (Fig. 3G). However, the phosphorylated TFE3 retained in the nucleus by calyculin alone or in combination with DT-061 did not affect ATF4 and CHOP protein expression. In fact, both DT-061 and Tg demonstrated the ability to promote ATF4 and CHOP expression and effectively transport them into the nucleus, however only DT-061 alone exhibited regulatory control over ATF4 and CHOP transcription through TFE3 dephosphorylation and nuclear shuttling. This demonstrates that PP2A activation is required for TFE3 dephosphorylation and subsequent transcriptional activation of ATF4 and CHOP. Given the robust effects of DT-061 in regulating TFE3 phosphorylation and nuclear shuttling, we examined changes in p-TFE3 localization in a panel of patient-derived xenograft tumors treated with DT-061 that we previously showed to significantly inhibit tumor growth in vivo [21]. We discovered that p-TFE3 levels decrease markedly in DT-061-treated tumors (5 mg/kg) compared to vehicle control treated tumors, while total levels remain unchanged, suggesting that p-TFE3 may serve as a biomarker for the therapeutic efficacy of DT-061. (Fig. 3H).

**Fig. 4:**
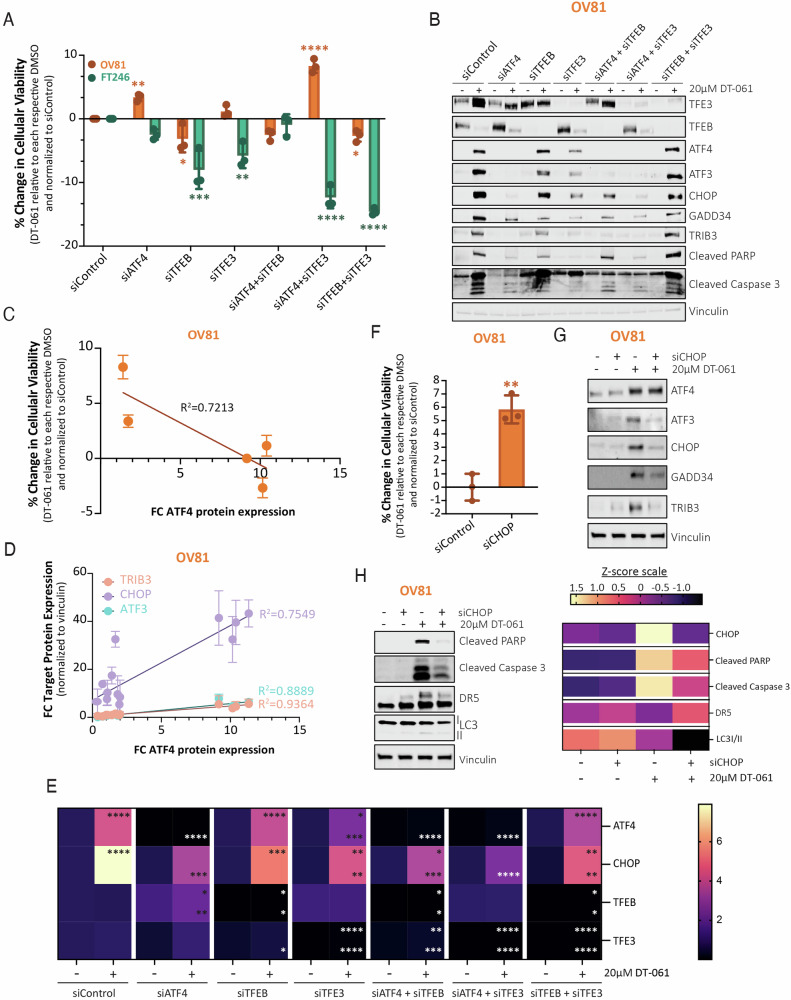
PP2A-mediated TFE3, ATF4, and CHOP activation is required for the DT-061-mediated cytotoxic effects. **A** siRNA experiments knocking down ATF4, TFEB, and TFE3 alone or in dual combination (i.e., ATF4 + TFEB, ATF4 + TFE3, and TFEB + TFE3) were performed in OV81 and FT246 to assess the dependency of DT-061-mediated cytotoxic effects on each of these proteins independently or in combination, evaluating recovery profiles in cell viability by cell titer glo analysis. Graphic representation was generated by calculating %change viability relative to siControl DT-061 treated condition for each respective cell line, after normalization to DMSO control. Data are presented as the mean ± SD (*n* = 3), (one-way ANOVA with multiple comparisons, comparing the mean of each column with the mean of siControl DT-061 treated column, *p < 0.05, **p < 0.01, ***p < 0.001, ****p < 0.0001). **B**–**E** siRNA conditions utilized in **A** were harvested for **B** protein and **E** RNA analysis. **B** Western blotting assessing the effect of each siRNA alone or in dual combination on the molecular changes of TFEB/3, ISR genes (ATF4, ATF3, CHOP, GADD34, and TRIB3), cell death (cleaved PARP, cleaved Caspase 3, and DR5) and autophagy (LC3) markers. Correlation analysis graph and *R*^2^ values comparing the expression of ATF4 with (**C**) % change viability or (**D**) each one of its downstream targets (CHOP, TRIB3, and ATF3) in OV81 after DT-061 treatment under RNAi conditions observed in (**A**, **B**). **E** qPCR analysis evaluating ATF4 and its downstream targets’ genetic expression in OV81 is represented as a bar graph. Data presented as the mean ± SD (*n* = 3) (one-way ANOVA with multiple comparisons, comparing the mean of each column with the mean of siControl DT-061 treated column for each respective target and cell line, ns > 0.05, *p < 0.05, **p < 0.01, ****p < 0.0001, top-relative to siControl DMSO, bottom-relative to its own si DMSO). **F** siCHOP cellular viability experiments in OV81 were pursued to assess dependency profiles of DT-061-mediated cytotoxic effects on CHOP protein expression and evaluate recovery in viability measured by cell titer glo analysis. Graph was generated by calculating %change viability relative to siControl DT-061 condition, after normalization to DMSO. Data presented as the mean ± SD (*n* = 3), (one-way ANOVA, *p < 0.05, **p < 0.01, ***p < 0.001, ****p < 0.0001). **G** Western blotting analysis in OV81 assessing the effect of siCHOP on the molecular signature downstream of ISR genes (ATF4, ATF3, CHOP, GADD34, and TRIB3), and **H** cell death (cleaved PARP, cleaved Caspase 3, and DR5) and autophagy (LC3) markers after treatment with DT-061 (left). Representation by heatmap of Z-score values obtained after western blot quantification using image J and normalization to Vinculin as the loading control (right). Calculated z-scores were obtained from protein quantification of three independent biological replicates.

To further confirm that the DT-061 mediated cytotoxic effects were induced by the PP2A-TFE3-ATF4 pathway, we performed siRNA experiments, knocking down ATF4, TFEB, and TFE3 alone or in dual combination (i.e., ATF4 + TFEB, ATF4 + TFE3, and TFEB + TFE3). Cell viability data (Fig. 4A) and death marker protein expression (Fig. 4B) show that the loss of ATF4, TFE3, or the combination of the two can significantly abrogate DT-061 effects on cancer cell death. Consistent with this data, the expression of ATF4 and its downstream targets, including ATF3, CHOP, and TRIB3 expression levels correlated with DT-061-mediated viability in cancer cells (Fig. 4C, D). Interestingly, ATF4, TFE3, and TFEB seem to play crucial roles in maintaining cellular balance and ensuring homeostasis in normal cells treated with DT-061, as any perturbation and combination thereof proved detrimental to viability of FT246 cells (Fig. 4A). Even though targeted inhibition of TFEB alone had no effect on DT-061-mediated protein induction of ATF4 or CHOP, RNA levels were significantly suppressed (Fig. 4B, E). Nonetheless loss of TFE3 alone or in combination with ATF4 significantly blunted the DT-061-induced upregulation of ATF4 and CHOP on both protein and RNA levels. Surprisingly, siATF4 at baseline decreased TFE3 levels, suggesting that ATF4 may also regulate TFE3 mRNA expression as a negative feedback mechanism, although no previous literature supports this claim. Finally, we determined that siRNA-mediated CHOP knockdown significantly rescued cellular viability in cancer cells upon DT-061 treatment (Fig. 4F) and reduced the expression of CHOP downstream transcriptional targets (Fig. 4G), while also attenuating the induction of cleaved PARP, cleaved Caspase 3, DR5, and LC3I/II expression (Fig. 4H).

In summary, these findings indicate that DT-061 mediates TFE3 dephosphorylation via PP2A, subsequentially promoting the transcriptional activation of ATF4 and CHOP that drives irreversible ISR phenotypes and triggers pro-death signals in cancer cells.

## Discussion

Targeting PP2A has emerged as an effective therapeutic strategy for the treatment of human cancers due to its unique capability in regulating multiple crucial cellular processes [38]. Intriguingly, despite their multifunctional properties in cellular contexts, PP2A modulators such as DT-061, have demonstrated pre-clinical promise by selectively targeting cancer cells for death while lacking adverse effects on normal tissues [21–28]. Prior research has consistently demonstrated DT-061’s favorable in vivo outcomes and tolerability, effectively reducing tumor burden; however, the mechanisms dictating such broad antitumor properties has remained unknown. In the current study, our aim focused on elucidating the intricate mechanisms governing the adaptation of non-transformed cells to chemical PP2A activation, thereby unveiling alternative non-canonical pathways that dictate cellular survival in response to this class of compounds. We have previously demonstrated the high tolerability profiles in non-malignant fallopian tube models to DT-061 in vitro after treatment with lower concentrations of DT-061 [21]. In the current study, the low-dose colony-forming assays have corroborated the capacity of normal cells to adapt and sustain a durable response, reprograming baseline homeostatic conditions to ensure survival under prolonged stress cues. Intriguingly, both malignant and non-malignant models displayed toxicity phenotypes when exposed to high DT-061 doses (20 µM) following 48 h of consecutive treatment. Subsequent washout studies that recapitulate metabolic clearance revealed that after removing DT-061, non-transformed cells can recover their viability, whereas HGSC cells are irreversibly committed to death. Our research reveals an intrinsic mechanistic vulnerability within cancer cells in the PP2A/TFE3 pathway. DT-061-mediated activation of this pathway triggers an irreversible ISR, resulting in the initiation of pro-death signals governed by ATF4 and CHOP activity, ultimately overpowering adaptive responses. In contrast, non-malignant tissues exhibit ISR plasticity, instigating adaptive homeostatic mechanisms that effectively uphold cellular survival during ISR activation induced by PP2A chemical modulators. This resilience is driven by ATF4 and CHOP-induced transcriptional and translational reprogramming, preventing irreversible ISR, and favoring pro-survival signals.

During cellular stress responses, the ISR rapidly and efficiently resolves cytotoxic and proteolytic stress favoring cell survival and homeostatic recovery mechanisms. However, sustained activation of these pathways results in the exacerbated activation of ATF4, a master regulator of cellular survival outcomes, thereby accumulating irreversible pro-death signals and determining deleterious cell fates [8, 10, 39, 40]. While PP2A’s role in regulating TFE3 to subsequently transcribe ATF4 and CHOP during ISR has been previously described [36], its involvement in governing ISR reversibility was never explored. Our studies propose a novel mechanism whereby chemical PP2A modulation chronically activates TFE3, consequently inducing ATF4 and CHOP expression and activity. This ultimately triggers an irreversible cellular stress response in cancer cells, which serves as a novel mechanism for preventing ISR plasticity in this context. We further uncovered that through the dephosphorylation and nuclear translocation of the transcription factor TFE3, DT-061 induces chronic ISR independent of PERK activation. Moreover, our results show that DT-061 modulation of PP2A regulates TFE3 to promote ATF4 expression and activity in both cancer and non-malignant cells, albeit distinctive downstream response mechanisms are activated, resulting in two distinct outcomes. In cancer cells, DT-061 treatment drives specific t-RNA charging signaling pathways to bias the translational machinery toward activating oxidative stress and ISR genes, such as ATF4, CHOP, DR5, and TRIB3, ultimately leading to an increase in autophagy, necroptosis, and apoptosis triggered by chronic and irreversible ISR. On the other hand, non-transformed cells’ priority is to restore normal translational and secretory abilities by shutting down pro-death pathways. Ultimately, our studies unveil that DT-061 initiates two parallel pathways: PERK activation and PP2A-TFE3-mediated induction of the ISR. Importantly, the phosphorylation of eIF2α alone was proved to be insufficient to promote a chronic and irreversible ISR state, requiring TFE3-mediated transcriptional activity to sustain prolonged ATF4 expression and activity.

Recently, selective chemical inhibition of PP2A has been shown to attenuate cellular stress response in plant cells treated with tunicamycin [41]. Nonetheless, the molecular mechanisms underlying PP2A’s regulation these stress mechanisms, and which specific heterotrimeric components are responsible to modulate cellular response to proteolytic and stress imbalances were not identified. Furthermore, the role of PP2A in regulating cellular stress-mediated responses in human cells are lacking, therefore requiring more thorough investigation. To date, literature shows that PP2A forms a complex with RACK1 to dephosphorylate and inactivate IRE1α during ER stress conditions in pancreatic β-cells [42]. Our studies are the first to propose a new role of PP2A in triggering irreversible ISR via TFE3-ATF4 transcriptional and translational expression regulation, selectively targeting cancer cells for death while normal cells adapt. Nonetheless, this manuscript does not fully explore the alternative mechanism dictating ATF4 protein synthesis, and the machinery recruited by PP2A to translate ATF4 mRNA under DT-061 treatment remains elusive. ATF4 mRNA has two upstream open reading frames (uORFs) [43, 44]. Under normal conditions, abundant eIF2-t-RNA tertiary complexes (TC) ensure continuous ribosomal translation until encountering the inhibitory uORF2, where translation halts [5, 43]. Conversely, during stress, canonical eIF2α phosphorylation reduces eIF2-t-RNA TC efficiency and Met-t-RNA delivery, enabling ribosomes to bypass uORF2. This facilitates the resumption of translation at the mORF, thereby promoting efficient ATF4 protein synthesis. Recently, a distinct mechanism has been proposed to regulate ATF4 mRNA translation independently of the eIF2 complex during ER stress conditions. Vasudevan et al. identified the non-canonical initiation factor eIF2D and DENR as essential factors for ATF4 translation during tunicamycin treatment [45], recruiting Met-t-RNA de novo and independently of eIF2α [46]. Although approximately 50% of human mRNA rely on leaky scanning and re-initiation processes for mORF recognition, only recently this process has been more carefully molecularly characterized [47–49]. Thus, its role in ATF4 translation in the context of DT-061 is warranted and must be explored in future studies.

Our research further indicates that DT-061 impairs the secretome in both cancer and non-malignant cells. Whereas FT cells recover from ISR by reprogramming overall translation to initiate adaptive homeostasis, HGSC cells irreversibly trigger the ISR and accumulate autolysosomes during DT-061-induced autophagy, leading to cell death. The proteomics studies revealed that DT-061 induces NRF2-mediated oxidative stress in cancer cells while repressing it in normal cells under the same conditions. Interestingly, NRF2, a strongly selective gene (DepMap), has been shown to transcriptionally regulate ATF4 levels under oxidative stress conditions [50], forming a complex with PP2A to induce autophagy, apoptosis, and ROS accumulation in other disease contexts [51]. Given the similarity between these findings and our DT-061 results, further investigation into the NRF2-PP2A complex formation and activity in DT-061-induced cellular stress and cytotoxicity is warranted.

Our data revealed that DT-061 leads to ATF4-mediated cell death, caused by the chronic accumulation of CHOP-dependent signaling cascades, including apoptosis, autophagy, and ROS. Interestingly, despite previous observation that DT-061 treatment resulted in a G_1_ cell-cycle arrest in HGSC [21]; its correlation with cellular stress and CHOP expression were not previously established. CHOP protein upregulation has been formerly shown to mediate G_1_ cell-cycle arrest by interacting with p21, ultimately resulting in apoptosis [52, 53]. This suggests CHOP’s possible involvement in the previously observed cell-cycle arrest profiles during DT-061 treatment, contributing to its cytotoxic effects in cancer cells. Additionally, DT-061 significantly induced specific ISR stress-mediated apoptotic markers, such as DR5, a downstream transcription target of CHOP [54–56]. Edagawa et al. has previously shown that the PERK-ATF4-CHOP pathway mediates DR4 and DR5 activation in an ATF3/CHOP-dependent manner when TP53 is functionally lost or deleted [54], a characteristic signature of numerous cancers Moreover, DR4/5 induction can also be regulated via the IRE1α/TRAIL arm of the UPR by interacting with phosphorylated C-Jun, forming a complex and regulates DR4/5 transcriptional activity [55]. Studies have shown that the CHOP-DR5 signaling triggers extrinsic apoptosis due to severe and chronic ROS accumulation [57–59], which also occurs during DT-061 treatment. Previous research has established that CHOP activated by cellular stress induces autolysosomes maturation, contributing to LC3II-dependent autophagy during early stages of stress while inhibiting autophagy-mediated granules that guarantee apoptosis in later stages [60, 61]. Such studies suggest CHOP plays a crucial role in the transitioning between autophagy and apoptosis when stress surpasses specific thresholds, possibly explaining distinct outcomes in cancer versus non-transformed cells post DT-061 exposure. Together, our results further validate these findings, confirming that DT-061-mediated PP2A modulation functionally activates these previously established pathways in a CHOP-dependent mechanism.

**Fig. 5:**
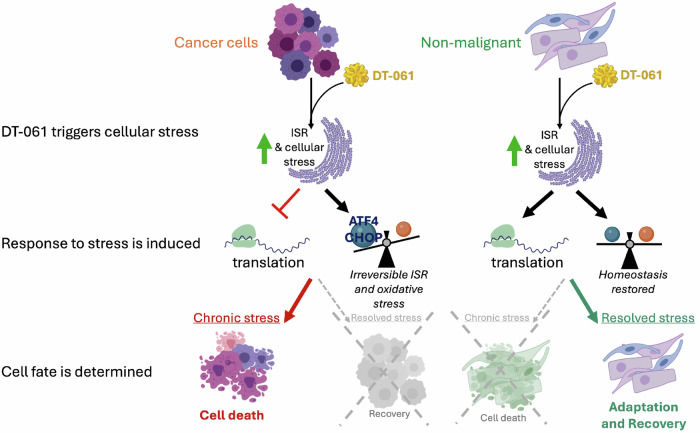
DT-061 exploits the cellular malignant state to induce differential ISR and determine cell survival. Schematic representing DT-061’s impact in cancer versus normal cellular survival fate. Our results show that upon DT-061 exposure, both cancer and non-malignant cells trigger stress signals, requiring adequate ER stress response mechanisms to restore homeostasis. OV81 cells are unable to respond to such cues efficiently and in a timely manner, activating chronic stress pathways, which result in cell death. On the other hand, normal cells are able to completely avert chronic and irreversible ER stress induction by activating efficient pro-survival feedback mechanisms, counteracting the activation of ER stress response pathways that allow for full recovery and cell survival.

In summary, our work indicates that DT-061, and potentially other chemical modulators of PP2A, regulate an alternative cellular stress response pathway, activating an irreversible ATF4-CHOP pro-death response in tumor cells but not in normal cells (Fig. 5). Our research lays a strong foundation for DT-061’s antitumor properties in multiple oncogenic contexts, thus explaining why it has widespread potent activities in a variety of cancers. In sum, our results reveal that PP2A is a master regulator of the ISR pathway through the regulation of TFE3, ATF4 and CHOP activity, highlighting the clinical promise of PP2A-modulating molecules due to their ability to selectively target cancer cells for death while minimizing toxicity to normal tissues.

## Materials and methods

### Protein digestion and TMT labeling

The samples underwent proteolysis and labeling using the TMT 10-plex method, following the manufacturer’s guidelines (ThermoFisher). After protein reduction with 5 mM DTT at 45 °C for 30 min, cysteine residues were alkylated using 15 mM 2-chloroacetamide at room temperature for 30 min. Protein precipitation involved adding 6 volumes of ice-cold acetone, followed by an overnight incubation at -20 °C. The resulting precipitate was centrifuged, and the pellet air-dried. The dried pellet was resuspended in 0.1 M TEAB, and overnight digestion with a trypsin/Lys-C mix (at a 1:25 protease-to-protein ratio, Promega) at 37 °C with continuous mixing in a thermomixer was carried out. The TMT 10-plex reagents were dissolved in 41 μl of anhydrous acetonitrile, and labeling was achieved by transferring the entire digest to the TMT reagent vial and incubating at room temperature for 1 h. The reaction was halted with 8 μl of 5% hydroxylamine and further incubated for 15 min. Labeled samples were combined and dried using a vacufuge. An offline fractionation of the merged sample (approximately 200 μg) into 8 fractions was conducted using a high pH reversed-phase peptide fractionation kit, as per the manufacturer’s instructions (Pierce, 84868). These fractions were then dried and reconstituted in 9 μl of a solution containing 0.1% formic acid and 2% acetonitrile in preparation for LC-MS/MS analysis.

### Global proteomics analysis

Raw mass spectrometry files were converted into open mzML format using the msconvert utility of the Proteowizard software suite [62]. MS/MS spectra were searched using the MSFragger database search tool [63] against an UniProt SwissProt protein sequence database appended with an equal number of decoy sequences [64]. For the analysis of whole proteome data, MS/MS spectra were searched using a precursor-ion mass tolerance of 20 ppm, fragment mass tolerance of 0.6 Da, and allowing C12/C13 isotope errors (0/1/2/3). Cysteine carbamidomethylation (+57.0215) and lysine TMT labeling (+229.1629) were specified as fixed modifications, and methionine oxidation (+15.9949), N-terminal protein acetylation (+42.0106), and TMT labeling of peptide N terminus and serine residues were specified as variable modifications. The search was restricted to fully tryptic peptides, allowing up to two missed cleavage sites. The search results were further processed using the Philosopher toolkit v4.5.1 [65]. First, MSFragger output files (in pepXML format) were processed using PeptideProphet [66] (with the high–mass accuracy binning and semi-parametric mixture modeling options) to compute the posterior probability of correct identification for each peptide to spectrum match (PSM). The resulting pepXML files from PeptideProphet (or PTMProphet) were then processed together to assemble peptides into proteins (protein inference) and to create a combined file (in protXML format) of high-confidence protein groups. Corresponding peptides were assigned to each group. The protein inference file and the individual PSM lists were further processed using the Philosopher filter command as follows. Each peptide was assigned either as a unique peptide to a particular protein group or assigned as a razor peptide to a single protein group that had the most peptide evidence. The protein groups assembled by ProteinProphet [67] were filtered to 1% protein-level false discovery rate (FDR) using the chosen FDR target-decoy strategy and the best peptide approach (allowing both unique and razor peptides) and applying the picked FDR strategy [68]. In each TMT plex, the PSM lists were filtered using a stringent, sequential FDR strategy, retaining only those PSMs with PeptideProphet probability of 0.9 or higher (which in these data corresponded to less than 1% PSM-level FDR) and mapped to proteins that also passed the global 1% protein-level FDR filter. For each PSM that passed these filters, MS1 intensity of the corresponding precursor-ion was extracted using the Philosopher label-free quantification module based on the moFF method [69] (using 10 p.p.m mass tolerance and 0.4 min retention time window for extracted ion chromatogram peak tracing). In addition, for all PSMs corresponding to a TMT-labeled peptide, TMT reporter ion intensities were extracted from the MS/MS scans (using 0.002 Da window) and the precursor ion purity scores were calculated using the intensity of the sequenced precursor ion and that of other interfering ions observed in MS1 data (within a 0.7 Da isolation window). All supporting information for each PSM, including the accession numbers and names of the protein/gene selected based on the protein inference approach with razor peptide assignment and quantification information (MS1 precursor-ion intensity and the TMT reporter ion intensities) was summarized in the output PSM files, one file for each TMT experiment. The tables were further processed using TMT-Integrator [70] to generate summary reports at the gene and protein level and, for phosphopeptide-enriched data, also at the peptide and modification site levels. In the quantitation step, TMT-Integrator used as input the PSM tables generated by the Philosopher as described above and created integrated reports with quantification across all samples at each level [71].

### Generation of patient-derived cell lines and cell culture

Patient samples were collected and PD cell lines were generated as previously described [21]. OV81 and CAOV3 cell lines were maintained in DMEM (ThermoFisher Scientific, 10-013-CV), PEO-1 and PEO-C4.2 in RPMI-1640 (Corning, 10-040-CV), and FT246 and FT237 in DMEM-F12 (ThermoFisher Scientific, 10-090-CV), all supplemented with 10% Fetal Bovine Serum (ThermoFisher Scientific, A3382001) and grown at 37 °C in 5% CO_2_. FT237 and FT246 were kindly provided by Dr. Drapkin and PEO cell lines by Dr. Taniguchi.

### Annexin-V and PI staining

Cells were plated overnight and treated the following day with vehicle control (DMSO) or DT-061 for 6 or 24 h. For each time point, media was collected, and cells were washed with cold 1× Phosphate Buffer Saline (PBS) (Fisher Scientific, SH3025601). Cells were then trypsinized, collected, and pelleted by centrifugation at 300 × *g* for 5 min at 4 °C. The supernatant was then aspirated, and the cell pellet was washed once more with cold PBS. Alexa Fluor 488 Conjugate Kit (Invitrogen, A13201) was used to stain cells at room temperature protected from light followed by flow cytometry analysis using a Bio-Rad ZE5 Cell Analyzer.

### LC3-dependent autophagy flux assay

The Premo^TM^ Autophagy tandem sensor RFP-GFP-LC3B kit (ThermoFisher Scientific, P36239) was utilized as a monitoring system of the autophagic flux in OV81 and FT246 cells LC3B-dependent. Cells were seeded in a white-sided with clear bottom 96-well plate (Corning, 3610) overnight with the BacMam 2.0 LC3B-FP reagent, at 37 °C in 5% CO_2_. The next day, cells were treated with either DMSO or DT-061 for 3, 6, and 9 h. The fluorescence signal was acquired using a plate reader.

### Reactive oxygen species accumulation

The MitoRos^TM^ 580 reagent (Fisher Scientific, 50-195-3583) was used to detect live ROS accumulation after 1, 3, 6, and 24 h of DMSO or DT-061 treatment. OV81 and FT246 cells were seeded in a white-sided with clear bottom 96-well plate overnight at 37 °C in 5% CO_2_. The next day, cells were treated with DMSO or DT-061. Thirty minutes prior to signal reading, cells were incubated with an equal volume of 2X mitoROS working solution. Hank’s Buffer with 20 mM HEPES (HHBS) was used to wash the cells three times and 100 µL was added at the final step. The fluorescence signal was obtained using a plate reader and the microscope pictures were taken using an Olympus IX70 fluorescence Microscope.

### Proliferation and colony-forming assays

To measure cellular viability, each cell line was seeded in either a 12-well (for 3-(4,5-dimethylthiazol-2-yl)-2,5-diphenyltetrazolium bromide assay (ThermoFisher Scientific, M6494)) or 96-well (for cell titer glo (Promega, G7572)) plate overnight to reach 70% confluency the next day. Cells were then treated with either DMSO (ThermoFisher Scientific, BP231-100), DT-061, Thapsigargin (EMD Millipore, T9033), PERK inhibitor II (EMD Millipore, GSK-2656157), or Calyculin A (Sigma, PHZ1044) and incubated for time points respective of each figure.

For colony-forming assays, cells were initially plated at a low density (500 and 100 cells/well for FT246 and OV81, respectively) in biological triplicates in a 6-well plate pre-coated with poly-D-lysine (Fisher Scientific, 08-774-270). After 48 h, cells were treated with either DMSO, DT-061, or Tg. Drug media was replenished every three days, except for the washout studies for which the media changing properties and frequencies are as described in each respective figure legend. On day 12, cells were fixed and stained using 1% crystal violet solution (Fisher Scientific, C581-25) and Image J software was used to quantify the number of colonies formed.

### Western blotting

Cells were harvested for protein isolation using 1X RIPA Lysis and Extraction Buffer (EMD Millipore, 20-188), 5% glycerol, and a cocktail of phosphatase (Thermo Fisher Scientific, A32957) and protease inhibitors (Thermo Fisher Scientific, A32955). Protein quantification of the extracts was performed using the Pierce BCA Protein Assay kit (Thermo Fisher Scientific, 23227). Samples were then prepared with protein concentrations ranging between 1 and 2 µg/µl, 1× LDS buffer (Thermo Fisher, NP0007), 2.5% of 2-Mercaptoethanol (Sigma-Aldritch, M6250-250ML), and 1× RIPA. Samples were then run on a 12% or 4–20% gradient SDS–polyacrylamide electrophoresis gel (BioRad, 4568045 or 4568095) at 70–200 V. Proteins were transferred onto a nitrocellulose membrane (Bio-Rad, 1704159) with the quick semi-wet transfer Trans-Blot Turbo machine. Membranes were blocked for 1 h in 5% non-fat milk (Thermo Fisher Scientific, 50488785) made with 1X Tris-Buffered Saline Tween20 (TBST) buffer (AMRESCO 10791-792). Primary antibodies were incubated in either 3% Bovine Serum Albumin (BSA) (Fisher Scientific, 22070008-3) or 5% milk diluted in 1X TBST and secondaries in 5% milk. Antibodies were purchased from Cell Signaling: BiP (3177), PERK (3192), p-eIF2α Ser51 (3597), t-eIF2α (2103), ATF4 (11815), ATF3 (33593), TRIB3 (43043), DR5 (8074), cleaved Caspase 3 (9661), cleaved PARP (9541), LC3I/II (12741), PP2A-Aα (2041), PP2A-C (2041); Santa Cruz: CHOP (sc-166682), Vinculin (sc-73614), GAPDH (sc-47724); Proteintech: GADD34 (10449-1-AP); Sigma-Aldrich: TFE3 (HPA023881); 5) Fortis Life Sciences: TFEB (A303-673); Abcam: Lamin B1 (ab133741), and EMD Millipore: Puromycin (MABE343).

### Gaussia luciferase

Gaussia Luciferase was utilized as a reporter assay to monitor the secretory pathway as previously reported by Badr et al. [31]. The plasmid was purchased through Dr. Tannous’ lab by Prolume/NanoLight. OV81 and FT246 cells were seeded on day 1 and treated on day 2 with DMSO, 20 µM DT-061, or 5 µg/ml of BFA for 3, 6, 12, and 24 h. Both the media and cell lysates were collected to detect Gaussia luciferase luminescence intensity as indirect measurements of secretion and overall protein expression, respectively. Studies were performed according to the protocol described by Pierce^TM^ Gaussia luciferase Flash Assay Kit (Thermo Fisher Scientific, 16158). Gausia luciferase protein was detected via western blotting (Invitrogen, PIPA1181) and RNA via qPCR analysis using the Gaussia luciferase forward 5′-ATCTGCCTGTCCCACATCAA -3′ and Reverse 5′-GTCCACACACAGATCGACCT-3′ primers.

### SUnSET protein synthesis monitoring

Nascent protein translation was measured utilizing the SUrface SEnsing of Translation (SUnSET), a radioactive free puromycin-based method previously described by Schmidt et al. [72]. Pulse-chase experiments were performed as follows: Cells were plated overnight at 37 °C in 5% CO_2_. The next day, cells were then treated with either DMSO or DT-061. One hour prior to harvesting, 10 min of 10 µg/ml puromycin (Sigma-Aldrich, P8833) incubation (pulse) was performed to label nascent polypeptide chains, followed by 50-min incubation with puromycin-free media containing either DMSO or DT-061 (chase). One wash of cold 1× phosphate buffer saline (PBS) (Fisher Scientific, SH3025601) was used in between the two phases. Cells were then harvested and puromycin labeling was measured via western blotting techniques.

### Quantitative reverse transcriptase polymerase chain reaction analysis

Total RNA was isolated from OV81 and FT246 cells (Norgen Biotek, 48400) for cDNA preparation (Thermo Fisher Scientific, 4387406). cDNA was amplified via PCR using a Roche Lightcycler II real-time PRC machine using gene-specific PCR primers and SYBR Green Master mix (Thermo Fisher Scientific, A46111). 18S was used as a reference gene with the following primer sets: Forward 5′-CATCCTTTACATCCTTCTGTCTGT-3′ and Reverse 5′-GGAAAGCAGACATTGACCTC AC-3′. ATF4: Forward 5′-GCACTTCAAACCTCATGGGTTCTC-3′ and Reverse 5′-GGCTCATACAGATGC CACTATC-3′. ATF3: Forward 5′-GCCATTGGAGAGCTGTCTTC-3’ and Reverse 5’-GGGCCATCT GGAACATAAGA-3’. CHOP: Forward 5’-CAGAACCAGCAGAGGTCACA-3′ and Reverse 5′-AGCT GTGCCACTTTCCTTTC-3′. GADD34: Forward 5′-CTTCTGCCTTGTCTCCAGGA-3′ and Reverse 5′-GAC GCCTCTCCTGAACGATA. TRIB3: Forward 5′-CCGTCTTGGGCCCTATGT-3′ and Reverse 5′-GT ACCAGCCAGGACCTCAGT-3′. TFEB: Forward 5′-CCTGGAGATGACCAACAAGCAG-3′ and Reverse 5′-TAGGCAGCTCCTGCTTCACCAC-3′. TFE3: Forward 5′-GATCATCAGCCTGGAGTCCAGT-3′ and Reverse 5′-AGCAGATTCCCTGACA CAGGCA-3′.

### Small interference RNA

siRNA molecules targeting human ATF4 (s1704), CHOP (s3997), TFE3 (s14030), and TFEB (s15495) were purchased from Thermo Fisher Scientific and diluted in the provided nuclease-free molecular grade water. Silencer Select Negative control (4390843) was used as the appropriate control. MISSION esiRNA targeting human PPP2R1A (EHU071351) was obtained from Sigma-Aldrich and diluted nuclease-free TE buffer (10 mM Tris-HCl, pH 8.0, 1 mM EDTA). Lipofectamine RNAiMAX (Thermo Fisher Scientific, 13778150) was used to perform all transfections.

### Nuclear and cytoplasmic protein extraction

NE-PER nuclear and cytoplasmic protein isolation was performed as recommended by the Thermo Scientific kit (78835). Each reagent was supplemented with a phosphatase (Thermo Fisher Scientific, A32957) and a protease inhibitor (Thermo Fisher Scientific, A32955) tablet for a complete extraction buffer preparation.

### Statistical methods

Statistical methods used in the current manuscript are described in detail in each respective figure legend. Fold change calculations, appropriate controls, and normalization approaches are further specified. All quantifications were statistically evaluated using PRISM software, except for global proteomics and RNA-sequencing.

## Supplementary information

Supplementary Material

Original Westerns

## Data Availability

All other data and materials supporting our findings can be made available from the corresponding authors upon request.
