# Supplementary material for: Integrated stress response plasticity governs normal cell adaptation to chronic stress via the PP2A-TFE3-ATF4 pathway
Source: Cell Death Differ. 2024 Sep 30;31(12):1761–75. doi: 10.1038/s41418-024-01378-3 (PMC11618521; doi:10.1038/s41418-024-01378-3)
Supplement: Supplementary file 1 — Supplementary Material [file 41418_2024_1378_MOESM1_ESM.pdf]

Supplemental Figure 1

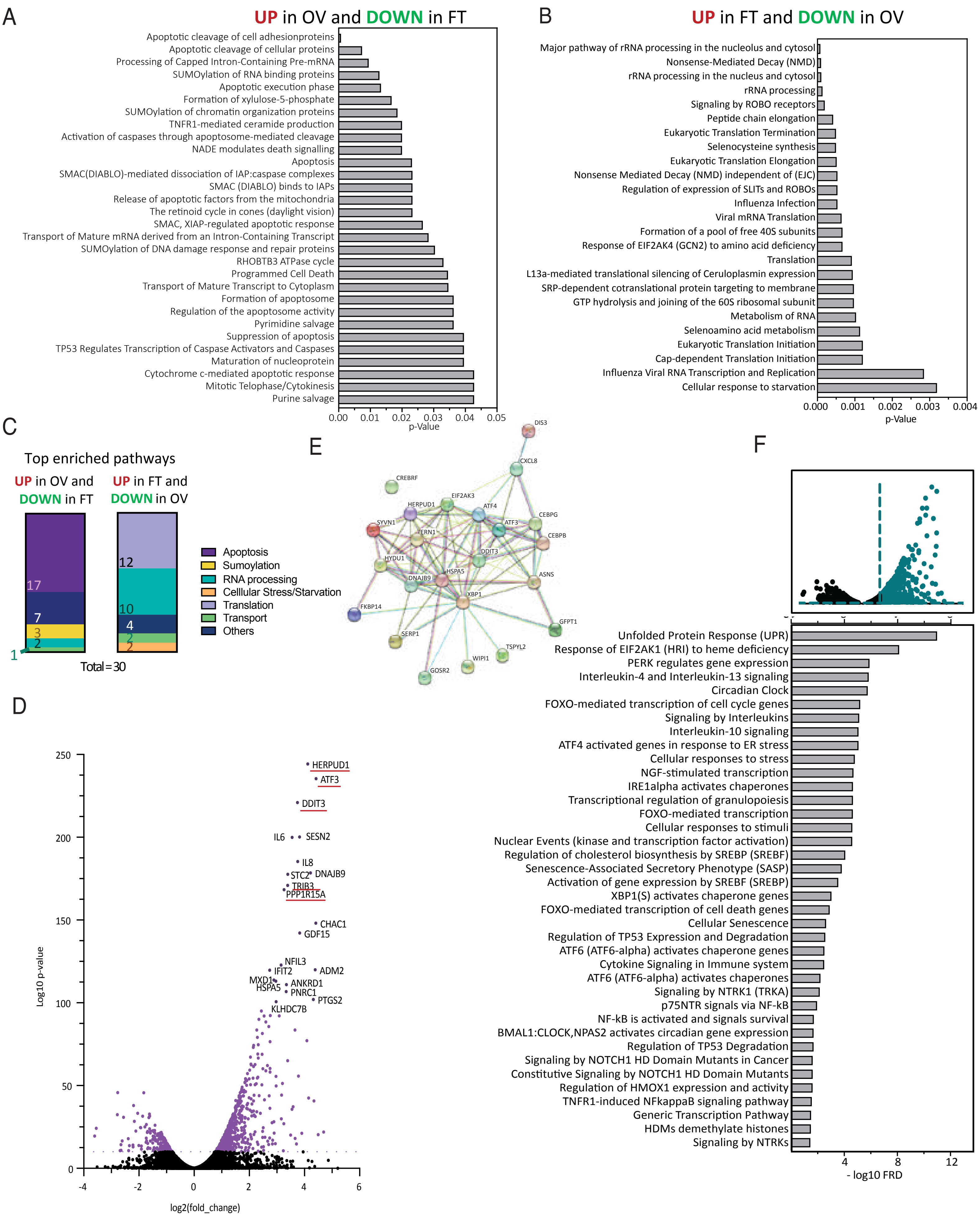

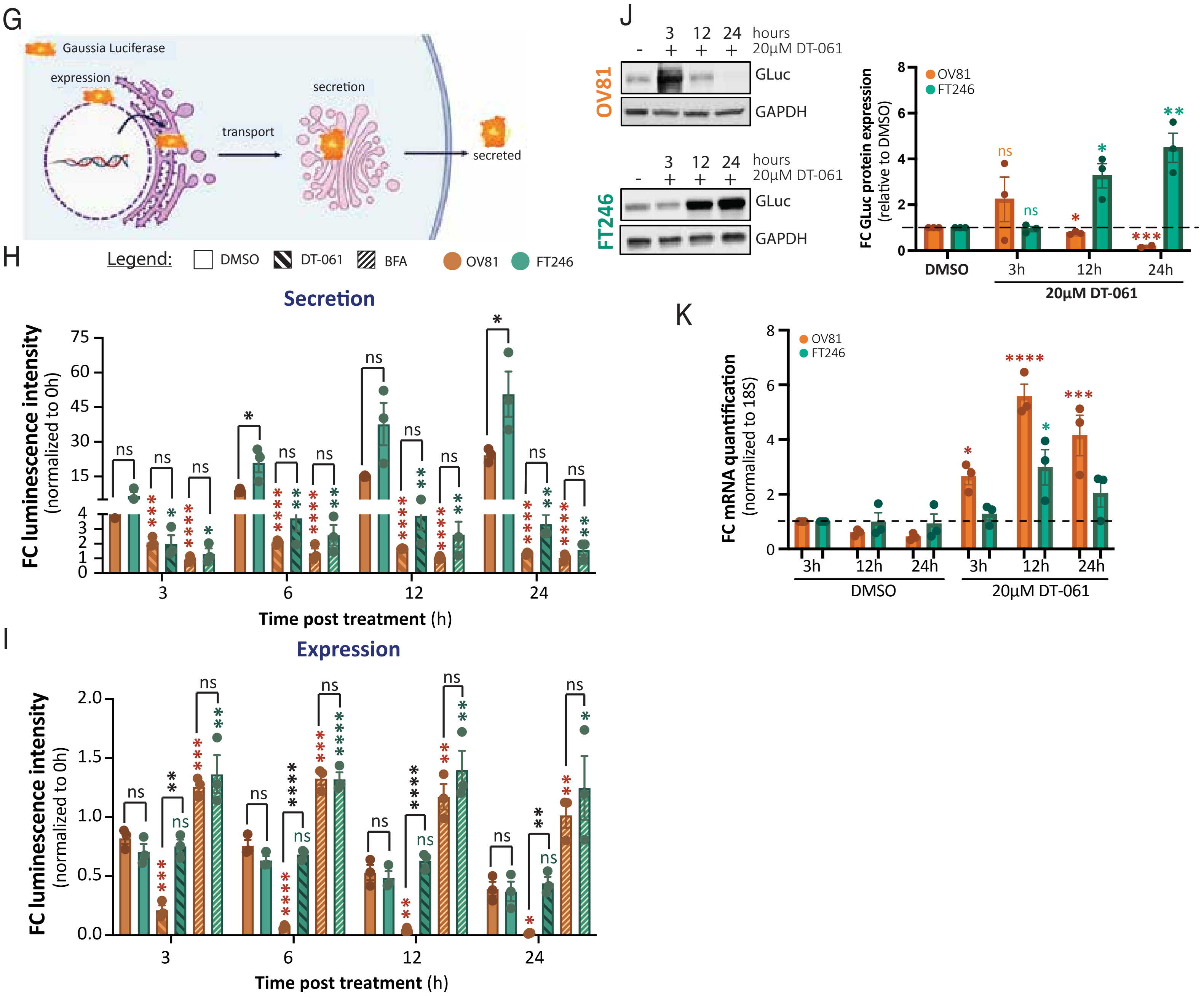
G
Gaussia Luciferase
expression
secretion
transport
secreted
H
Legend:
DMSO
DT-061
BFA
OV81
FT246
Secretion
FC luminescence intensity
(normalized to 0h)
Time post treatment (h)
I
Expression
FC luminescence intensity
(normalized to 0h)
Time post treatment (h)
J
hours
20µM DT-061
OV81
GLuc
GAPDH
FT246
GLuc
GAPDH
FC GLuc protein expression
(relative to DMSO)
DMSO
3h
12h
24h
20µM DT-061
K
OV81
FT246
FC mRNA quantification
(normalized to 18S)
3h
12h
24h
DMSO
20µM DT-061

Supplemental Figure 2

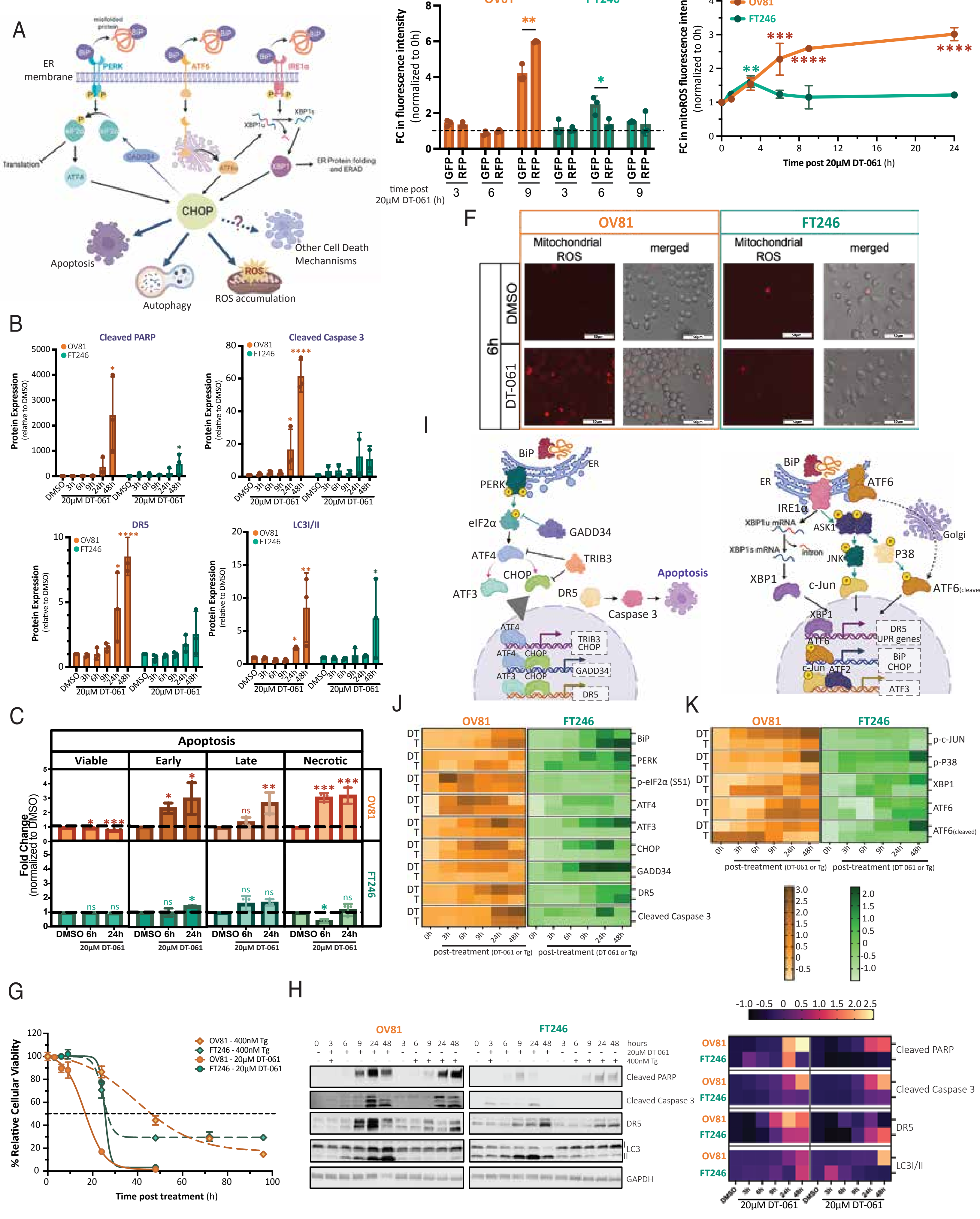

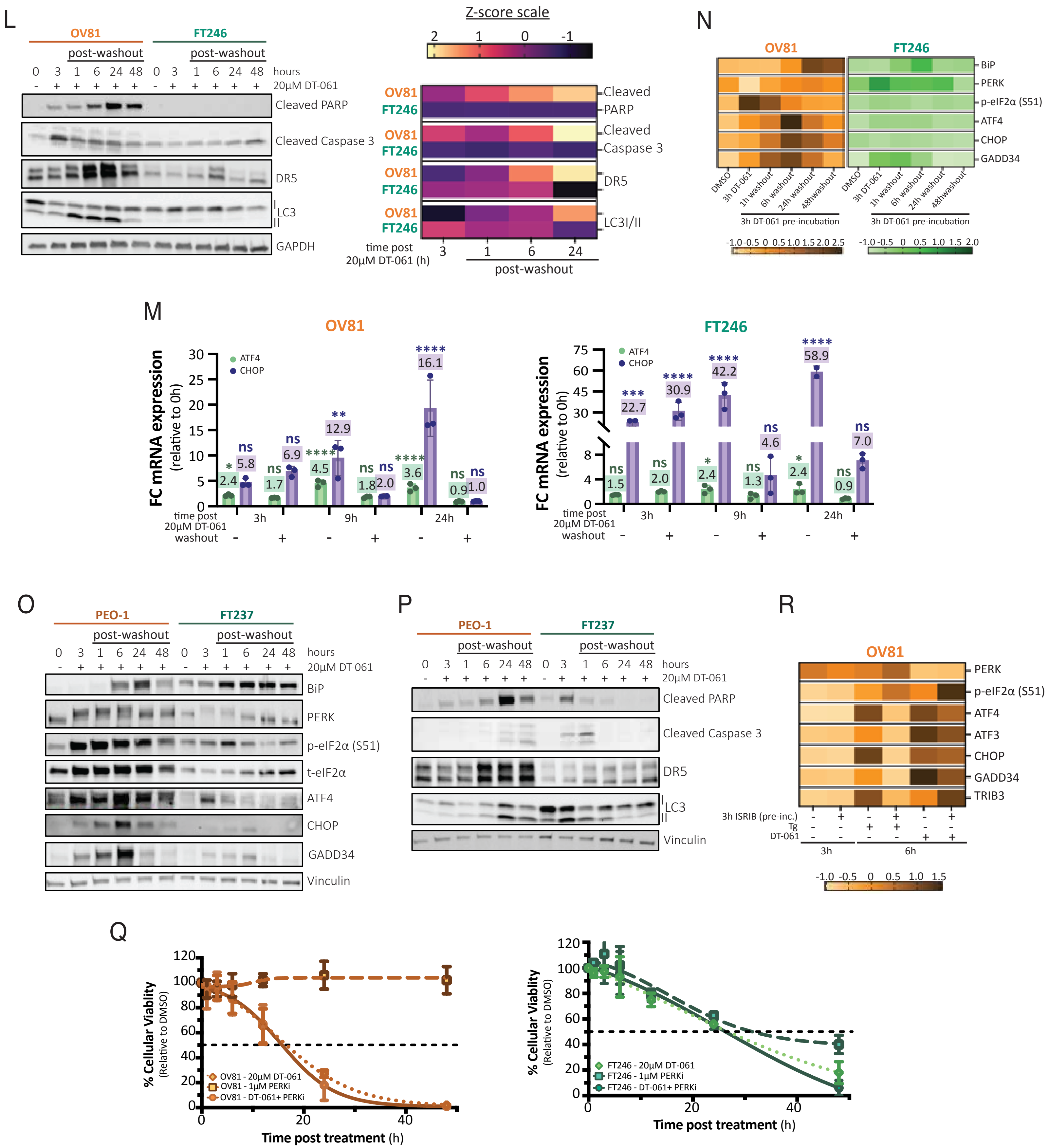

L
OV81
FT246
post-washout
post-washout
0 3 1 6 24 48 0 3 1 6 24 48 hours
- + + + + + - + + + + + 20µM DT-061
Cleaved PARP
Cleaved Caspase 3
DR5
LC3
I
II
GAPDH
Z-score scale
2 1 0 -1
OV81
FT246
Cleaved PARP
Cleaved Caspase 3
DR5
LC3I/II
time post 20µM DT-061 (h)
3 1 6 24
post-washout
N
OV81
FT246
BiP
PERK
p-eIF2α (S51)
ATF4
CHOP
GADD34
DMSO
3h DT-061
1h washout
6h washout
24h washout
48hwashout
3h DT-061 pre-incubation
-1.0 -0.5 0 0.5 1.0 1.5 2.0 2.5
-1.0 -0.5 0 0.5 1.0 1.5 2.0
M
OV81
FC mRNA expression
(relative to 0h)
ATF4
CHOP
2.4 5.8 1.7 6.9 4.5 12.9 1.8 2.0 3.6 16.1 0.9 1.0
time post 20µM DT-061
3h 9h 24h
washout - + - + - +
FT246
1.5 22.7 2.0 30.9 2.4 42.2 1.3 4.6 2.4 58.9 0.9 7.0
O
PEO-1
FT237
post-washout
post-washout
0 3 1 6 24 48 0 3 1 6 24 48 hours
- + + + + + - + + + + + 20µM DT-061
BiP
PERK
p-eIF2α (S51)
t-eIF2α
ATF4
CHOP
GADD34
Vinculin
P
PEO-1
FT237
post-washout
post-washout
0 3 1 6 24 48 0 3 1 6 24 48 hours
- + + + + + - + + + + + 20µM DT-061
Cleaved PARP
Cleaved Caspase 3
DR5
LC3
I
II
Vinculin
R
OV81
PERK
p-eIF2α (S51)
ATF4
ATF3
CHOP
GADD34
TRIB3
3h ISRIB (pre-inc.) - + - + - +
Tg - - + + - -
DT-061 - - - - + +
3h 6h
-1.0 -0.5 0 0.5 1.0 1.5
Q
% Cellular Viablity
(Relative to DMSO)
OV81 - 20µM DT-061
OV81 - 1µM PERKi
OV81 - DT-061+ PERKi
Time post treatment (h)
FT246 - 20µM DT-061
FT246 - 1µM PERKi
FT246 - DT-061+ PERKi
Time post treatment (h)

Supplemental Figure 3

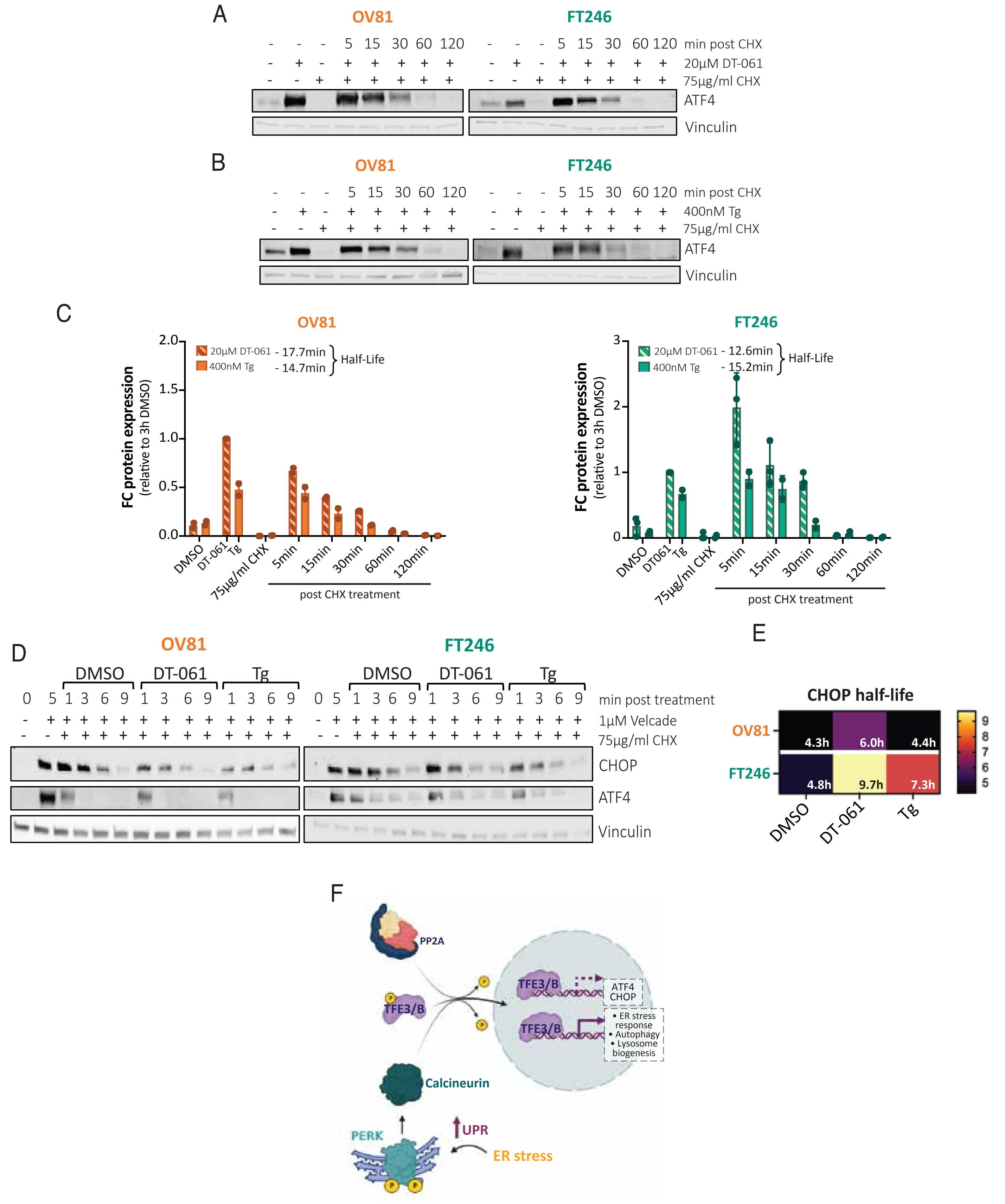

Supplementary Figure Legends

Supplemental Figure 1 – Global Proteomics and RNA-Sequencing Reveal Unique Signature Traits Specifically Induced in Cancer Cell Lines Upon DT-061 Treatment. A) and B) Detailed analysis of ***Fig. 1B*** including all pathways significantly induced with DT-061 treatment having opposite trends in malignant versus non-transformed lines. Data represented in order of significance with the smallest p-value listed at the top and separated by A) upregulated in OV and downregulated in FT or B) vice-versa. C) Top enriched pathways categorized by overall functional relevance and pathway clustering. Data separated by upregulated in OV and simultaneously downregulated in FT (left) or vice-versa (right). D) – F) RNA-seq results using H358 lung cancer cell line after DT-061 exposure for 24 hours were analyzed in detail. D) Volcano plot enriching for targets upregulated in DT-061 comparatively to DMSO (>0 values) or downregulated (>0 values). Data presented using $\log_2$ fold change for the x-axis and $\log_{10}$ p-value for the y-axis to evaluate statistical significance. Genes underlined with the red color are established protein targets of the ER stress response pathways previously identified in the global proteomics studies from ***Fig. 1A*** and ***Suppl. Fig. 1A*** as significantly activated in DT-061-treated conditions. E) STRING analysis evaluating the relationship and level of interaction among the topmost induced target hits after DT-061 exposure. F) Volcano plot categorizing the most significantly induced pathways using Reactome Pathway Analysis tools. Data represented in order of significance with the smallest false discovery rate (FDR) listed at the top. G) Schematic representing the expression and secretion cycle of Gaussia luciferase, depicting each important step for its efficient detection. This system was utilized as a reporter assay to monitor the secretory pathway and measure ER stress as previously described by Badr *et al.*(31). H) and I) Fold change luminescence intensity relative to 0h DMSO. H) Media was collected at 3, 6, 12, and 24 hours after treatment with DMSO, DT-061, or BFA as an indirect measurement of secretion ability. Data presented as the mean $\pm$ SD (n=3), (one-way ANOVA with multiple comparisons, comparing the mean of each column with the mean of DMSO control column, ns > 0.05, *p < 0.05, **p < 0.01, ***p < 0.001, ****p < 0.0001). I) Fold change luminescence intensity relative to 0h DMSO. Cell lysates were collected at 3, 6, 12, and 24 hours after treatment with DMSO, DT-061, or BFA as an indirect measurement

of protein expression. Data presented as the mean ± SD (n=3), (one-way ANOVA with multiple comparisons, comparing the mean of each column with the mean of DMSO control column, ns > 0.05, *p < 0.05, **p < 0.01, ***p < 0.001, ****p < 0.0001). F) Protein analysis of Gaussia luciferase expression was performed via western blotting (left), with its respective quantification represented (right). J) and K) Cells were collected after 3, 12, and 24 hours of DMSO or DT-061 treatment for J) protein and K) RNA analysis in both OV81 (orange) and FT246 (green) cell lines. Gaussia Luciferase mRNA expression in OV81 and FT246 was quantified using qPCR. Fold change mRNA expression levels were plotted relative to 3 hour DMSO after normalization to 18S housekeeping gene. Data presented as the mean ± SD (n=3), (one-way ANOVA with multiple comparisons, comparing the mean of each column with the mean of DMSO control column, ns > 0.05, *p < 0.05, ***p < 0.001, ****p < 0.0001).

Supplemental Figure 2 – DT-061-Mediated Cellular Survival Decision-Making is Molecularly Dependent on the Irreversible Activation of the ISR Pathway. A) Schematic of the three main arms of the Unfolded Protein Response (UPR) - PERK, IRE1α , and ATF6 – activating pro-apoptotic ER stressed responses. Upon prolonged unresolved stress and inability to restore cellular homeostasis, CHOP-dependent anti-survival pathways are activated, including apoptosis, autophagy, ROS accumulation, and other mechanisms of cell death. B) Western blotting quantification from ***Fig. 1C*** represented as a bar graph seeking to evaluate the expression of cell death, apoptosis, and autophagy markers in OV81 and FT246 after treatment with DT-061. Data quantified using Image J and presented as the mean ± SD (n=3), (one-way ANOVA with multiple comparisons, comparing the mean value for each column with the mean of DMSO control for its respective cell line OV81 (orange) and FT246 (green), *p < 0.05, **p < 0.01, ****p < 0.0001). C) Annexin V and PI staining quantifying viable cells (Annexin V- and PI-), in early (Annexin V+ and PI-), late (Annexin V+ and PI+) or necrotic (Annexin V- and PI+) states after DT-061 treatment for 6 and 24 hours. Results for OV81 are graphed in orange while FT246 in green. Data presented as the mean ± SD (n=3), (one-way ANOVA with multiple comparisons, comparing the mean of each column with the mean of DMSO control column for its respective time point and cell line, ns > 0.05, *p < 0.05, ***p < 0.001). D) Premo^{TM} Autophagy Tandem Sensor RFP-GFP-LC3B was used

to detect the expression of LC3II post 3, 6, and 9 hours of DT-061 exposure. RFP expression correlates with late maturation and pH resistant vesicles (=autolysosomes) while GFP represents premature phagosomes sensitive to pH changes (=autophagosomes). Data presented as the mean $\pm$ SD (n=3), (unpaired Student T-tests, comparing RFP expression relative to GFP for each individual time point and respective cell line, *p < 0.05, **p < 0.01). E) mitoROS was used to detect Reactive Oxygen Species (ROS) accumulation in cells after DT-061 exposure over time (1, 3, 6, 9, and 24 hours). Obtained results were graphed measuring the fold change of fluorescence intensity under DT-061 conditions normalized to its respective DMSO control for each time point in OV81 (orange) and FT246 (green). Data presented as the mean $\pm$ SD (n=3), (unpaired Student T-tests, comparing DT-061 treatment relative to 0h time point, **p < 0.01, ***p < 0.001, ****p < 0.0001). F) Immunofluorescent microscopy pictures representative of the 6 hour time point quantified in (E). Red signal – mitoROS dye incorporated by mitochondria that are positive for ROS activity. Merged – red fluorescent signal and bright field pictures of the same area overlapped. G) OV81 and FT246 cells were treated with 20µM of DT-061 or 400nM of Tg and cell viability was measured over time using cell titer glo. Response to treatment was evaluated in cancer versus non-malignant models, showing their ability to activate adaptive mechanisms (when treated with Tg) or yield to chronic stress being unable to survive (DT-061 treatment) during short but extreme conditions of stress. Data presented as the mean $\pm$ SD (n=3). H) Western blotting analysis evaluating the expression of cell death, apoptosis, and autophagy markers (cleaved PARP, cleaved Caspase 3 and DR5, and LC3I/II, respectively) in OV81 and FT246 after treatment with DT-061 or Tg over the course of 48 hours (left). Z-score values after western blotting quantification using image J and normalization to Vinculin as the loading control were plotted as a heatmap. Calculated z-scores were obtained from protein quantification of three independent biological replicates. I) Schematics of the UPR pathways represented in the western blotting analysis from ***Fig. 1E*** and ***1F,*** respectively, represented in the order of their activation and with their protein-protein interaction partners. J) and K) Western blotting analysis from J) ***Fig. 1E*** (PERK) and K) and ***1F*** (IRE1α, and ATF6) were quantified using Image J and represented in a z-score heatmap. Each condition was normalized to its respective Vinculin loading control. The calculated z-scores were obtained from protein quantification of three independent biological

replicates (legend: S – DT-061, T – Tg). L) Western blotting analysis evaluating the molecular mechanism and profiles of DT-061 post washout versus no-wash downstream ISR stress-mediated cell death and autophagy markers (left). Representation by heatmap of Z-score values was obtained after western blot quantification using image J and normalization to housekeeping (right) for each cell line and time point. Calculated z-scores were obtained from protein quantification of three independent biological replicates. M) mRNA from washout and no-wash experiments were extracted and qPCR analysis of ATF4 and CHOP expression levels was performed for OV81 (left) and FT246 (right). Data presented as the mean $\pm$ SD (n=3) (one-way ANOVA with multiple comparisons, comparing the mean of each column with the mean of DMSO control column for its respective target and cell line, ns > 0.05, *p < 0.05, **p < 0.01, ****p < 0.0001). N) Western blotting analysis evaluating the molecular signatures induced by DT-061 post-washout versus no-wash from in ***Fig. 1I*** were quantified and normalized relative to DMSO control using Image J. Representation by heatmap of z-score values is plotted after normalization to GAPDH housekeeping control. Calculated z-scores were obtained from protein quantification of three independent biological replicates. O) and P) Experiment from ***Fig. 1I*** was repeated using a HGS malignant (PEO-1) and non-transformed fallopian tube (FT237) cell lines to evaluate if the molecular signatures observed with DT-061 in washout conditions were reproducible in other ovarian/fallopian tube models. O) The PERK pathway and its downstream targets as well as P) ISR-mediated cell death and autophagy markers were evaluated via western blotting analysis. Q) Pre-incubation with a small molecule inhibitor of PERK phosphorylation and subsequent activity was tested to evaluate its impact on DT-061 cytotoxic effects in OV81 (left) and FT246 (right) cells. Cell viability was measured after 24 hours of treatment with 20µM of DT-061, 1µM of PERKi, or the combination of DT-061 + PERKi using cell titer glo. Data presented as the mean $\pm$ SD (n=3). R) Quantification of the western blotting analysis represented in ***Fig. 2D***. A small molecule inhibitor of the integrated stress response (ISRB) pathway was used to assess DT-061's dependency on p-eIF2α and its subsequent inhibition of cap-dependent translation for the translational regulation of ATF4 and CHOP proteins. The z-score values were plotted as a heatmap after quantification and normalization to respective Vinculin loading control. Calculated z-scores were obtained from protein quantification of three independent biological replicates.

Supplemental Figure 3 – DT-061 Affects the Stability of ATF4 and CHOP Proteins. OV81 and FT246 cells were preincubated with A) 20μM of DT-061 or B) 400nM of Tg for 3 hours followed by cycloheximide (CHX) treatment to determine ATF4 half-life under each treatment condition. Western blotting analysis was performed to measure ATF4 protein expression over time after active protein translation processes were halted by CHX. C) The decay rate was measured by quantifying ATF4 protein expression levels from A) and B) using Image J. ATF4 half-life for each treatment condition was calculated using PRISM and represented in the graph. ATF4 stability was revealed to remain relatively unchanged after DT-061 and Tg treatments, in both HGSC and non-transformed FT cells, only ranging 3 minutes between conditions. D) and E) OV81 and FT246 cells were preincubated with velcade proteasome inhibitor for 5 hours to stop protein degradation, thus accumulating overall protein expression and increasing stability. Velcade media was then removed and replaced with fresh media containing either DMSO, 20μM of DT-061, or 400nM of Tg in combination with CHX to halt new protein translation. D) Western blotting analysis was performed to measure CHOP's protein levels under each of these treatment conditions and E) their quantification was plotted in PRISM to calculate CHOP half-life. DT-061 increases CHOP stability in both OV81 and FT246 by 2 and 4 hours, respectively while Tg only increased CHOP half-life in FT246 by 2.5 hours, the latter possibly explaining Tg's toxicity effects previously observed in non-malignant tissues in ***Fig. 2G***. F) Schematic representing the mechanism by which TFEB and TFE3 are central components of the ER and integrated stress response. TFEB/3 are regulated and activated in the cytoplasm for nuclear transport, where they can act as transcription factors and activate ER stress responses, mTOR-dependent autophagy, and lysosome biogenesis genes. Two different phosphatases have been established to dephosphorylate TFEB/3 in the cytoplasm – Calcineurin (UPR-dependent) and PP2A (UPR-independent) – contingent on the type of stress and cellular context. Schematic designed in biorender.
