# Supplementary material for: Integrated stress response plasticity governs normal cell adaptation to chronic stress via the PP2A-TFE3-ATF4 pathway
Source: Cell Death Differ. 2024 Sep 30;31(12):1761–75. doi: 10.1038/s41418-024-01378-3 (PMC11618521; doi:10.1038/s41418-024-01378-3)

Original Western Blotting Images Presented in Fig. 1-4 and Supplementary Fig. 1-3

Fig. 1A

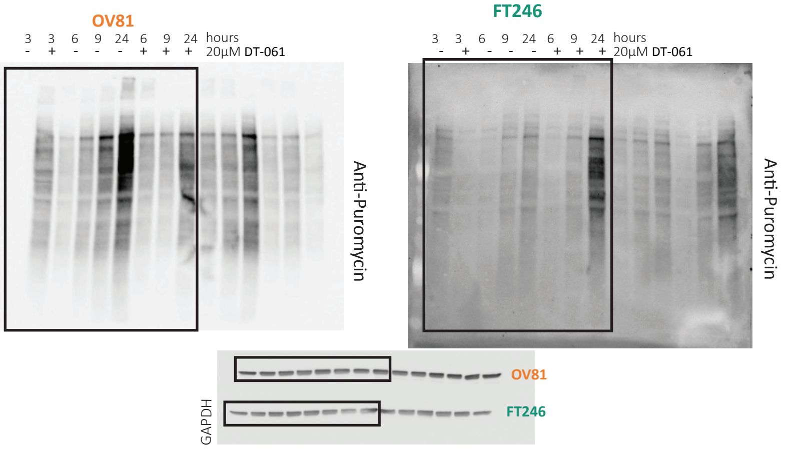

Fig. 1C

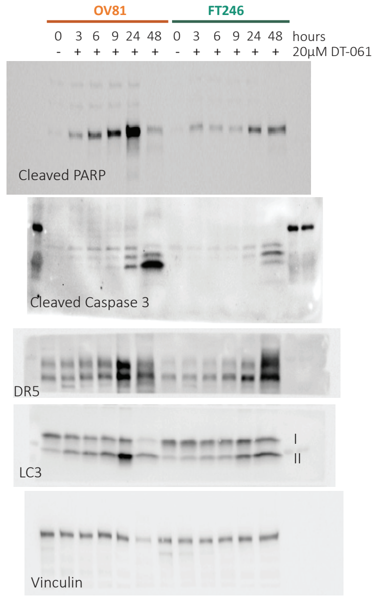

Fig. 1E

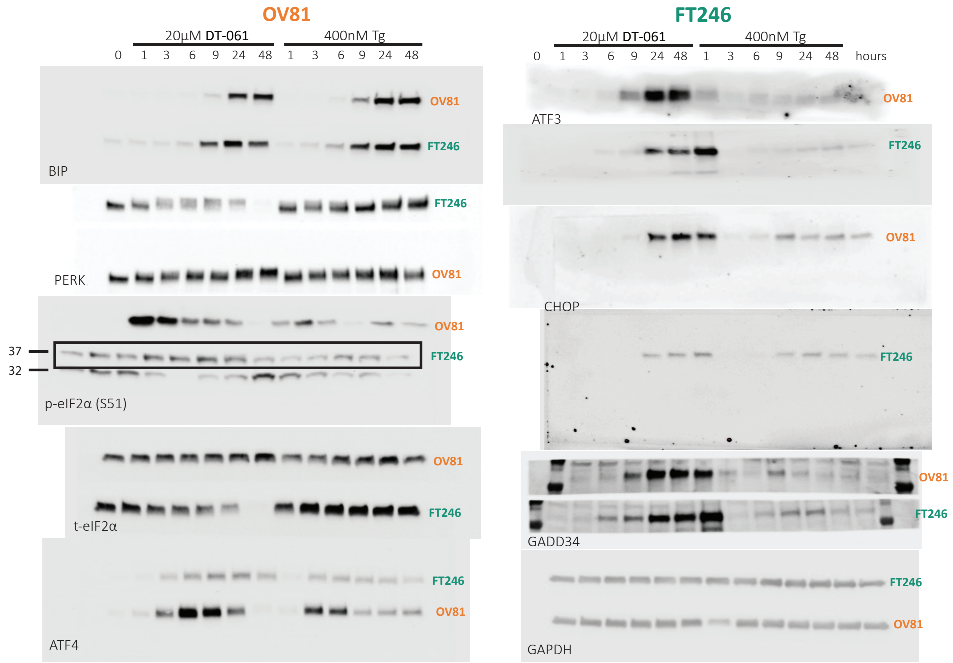

Fig. 1I

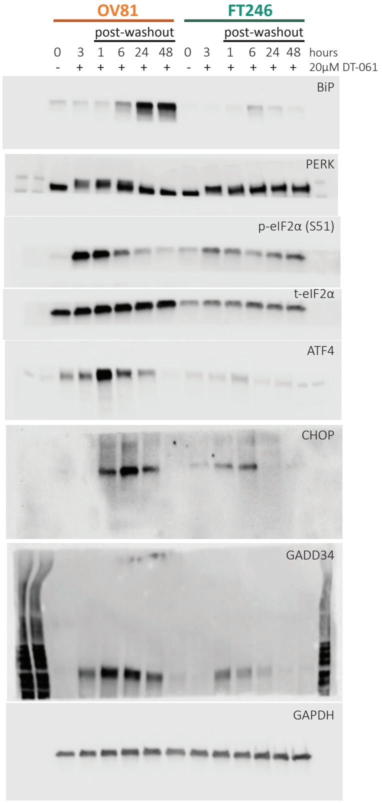

Fig. 1F

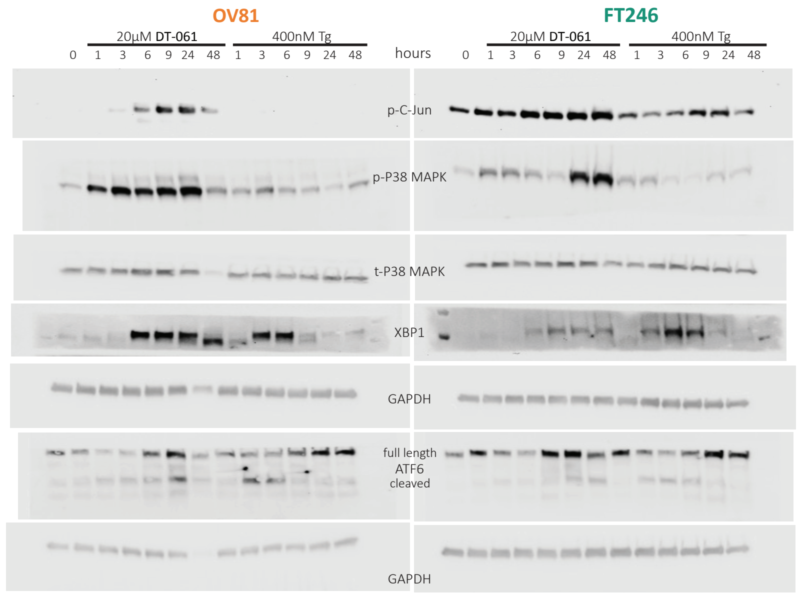

Fig. 2A

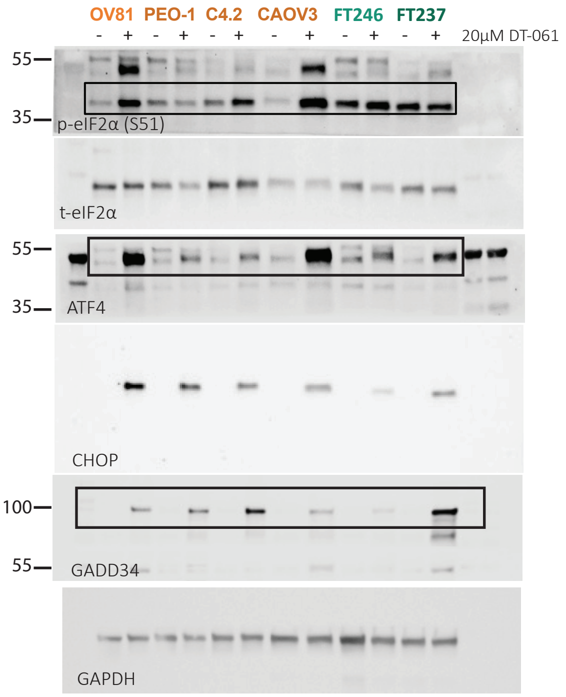

Fig. 2D

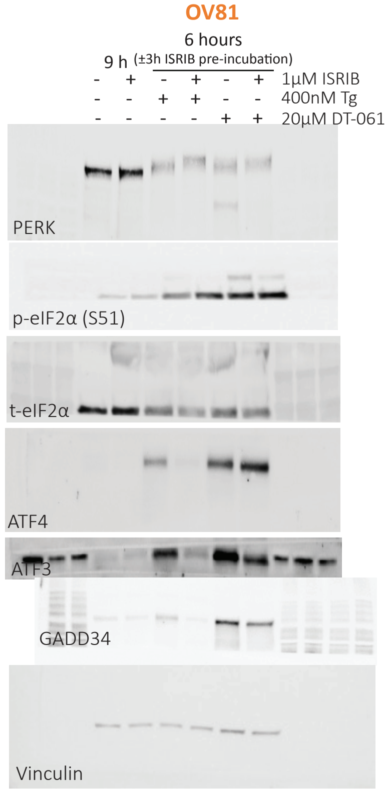

Fig. 2E

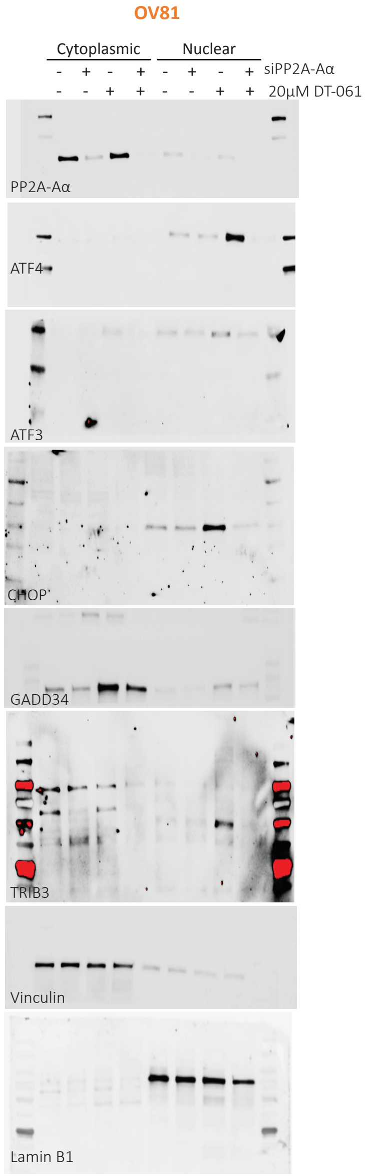

Fig. 2C

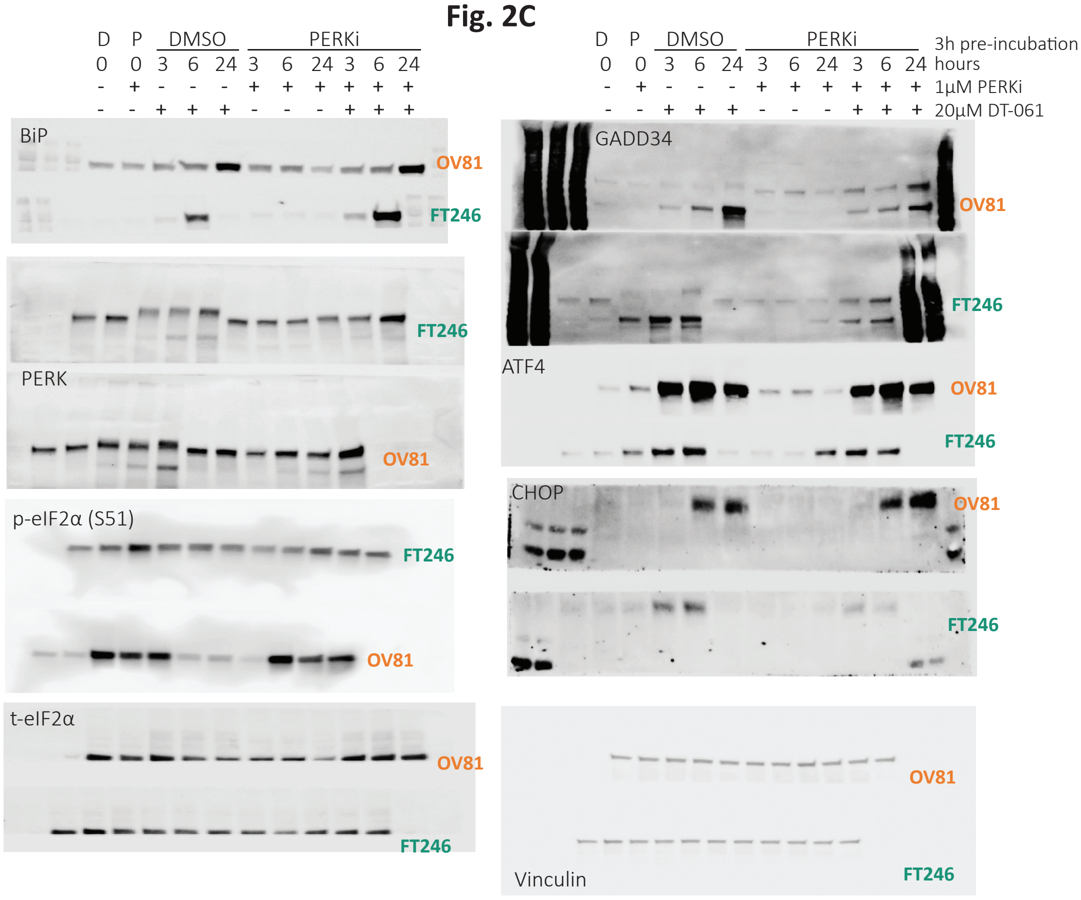

Fig. 2F

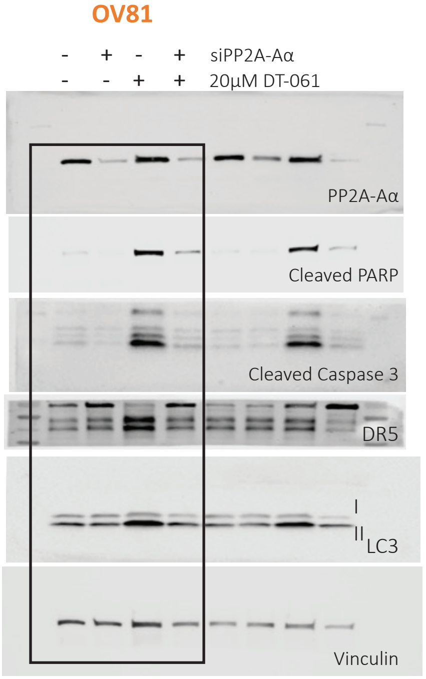
OV81
- + - + siPP2A-Aα
- - + + 20μM DT-061
PP2A-Aα
Cleaved PARP
Cleaved Caspase 3
DR5
I
II
LC3
Vinculin

Fig. 2G

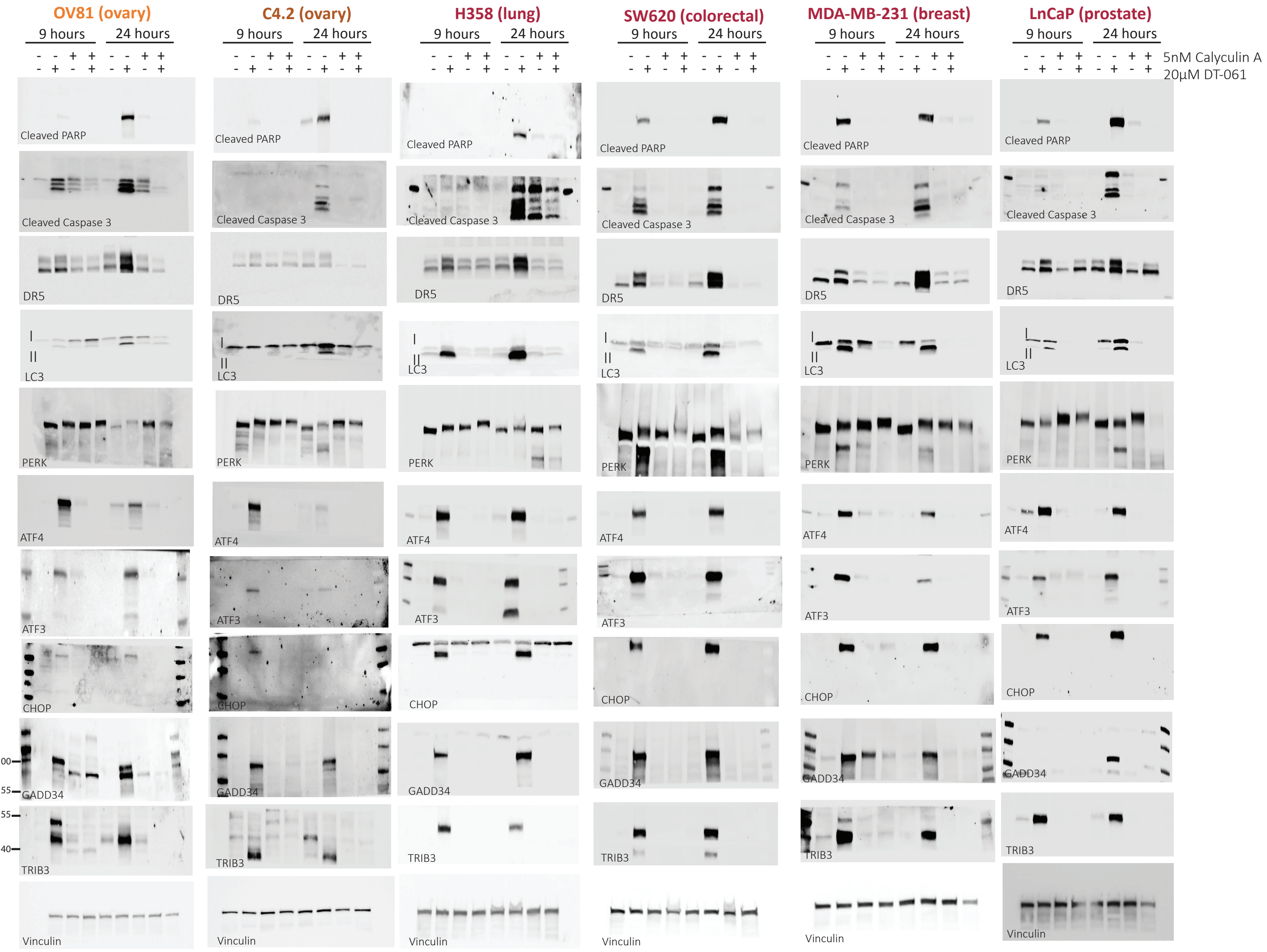
OV81 (ovary)
C4.2 (ovary)
H358 (lung)
SW620 (colorectal)
MDA-MB-231 (breast)
LnCaP (prostate)
9 hours
24 hours
5nM Calyculin A
20μM DT-061
Cleaved PARP
Cleaved Caspase 3
DR5
I
II
LC3
PERK
ATF4
ATF3
CHOP
GADD34
TRIB3
Vinculin
00
55
55
40

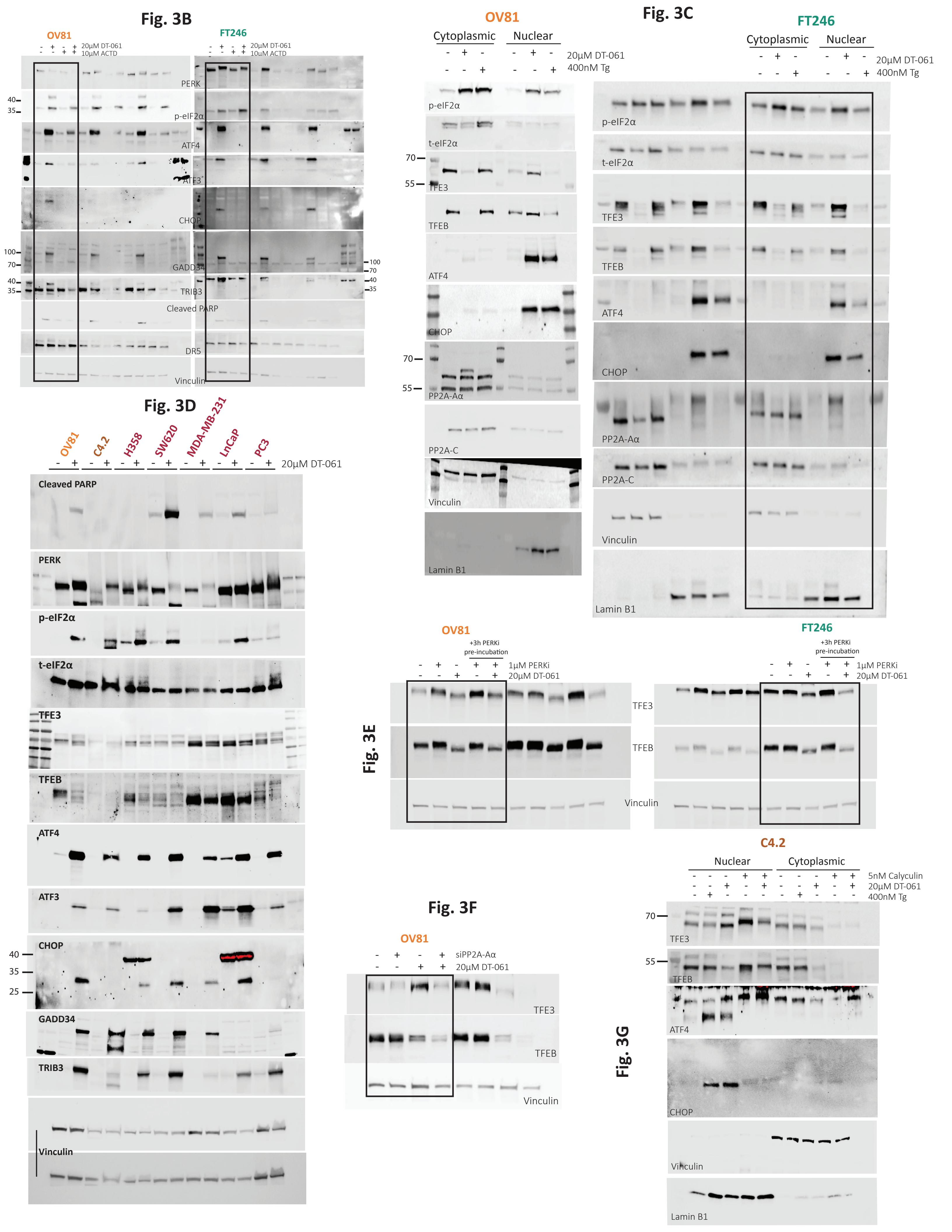

Fig. 3B
OV81
FT246
20µM DT-061
10µM ACTD
PERK
p-eIF2α
ATF4
ATF3
CHOP
GADD34
TRIB3
Cleaved PARP
DR5
Vinculin
40
35
100
70
Fig. 3C
OV81
FT246
Cytoplasmic
Nuclear
20µM DT-061
400nM Tg
p-eIF2α
t-eIF2α
TFE3
TFEB
ATF4
CHOP
PP2A-Aα
PP2A-C
Vinculin
Lamin B1
70
55
Fig. 3D
OV81
C4.2
H358
SW620
MDA-MB-231
LnCaP
PC3
20µM DT-061
Cleaved PARP
PERK
p-eIF2α
t-eIF2α
TFE3
TFEB
ATF4
ATF3
CHOP
GADD34
TRIB3
Vinculin
40
35
25
Fig. 3E
OV81
FT246
+3h PERKi pre-incubation
1µM PERKi
20µM DT-061
TFE3
TFEB
Vinculin
Fig. 3F
OV81
siPP2A-Aα
20µM DT-061
TFE3
TFEB
Vinculin
Fig. 3G
C4.2
Nuclear
Cytoplasmic
5nM Calyculin
20µM DT-061
400nM Tg
TFE3
TFEB
ATF4
CHOP
Vinculin
Lamin B1
70
55

Fig. 4B

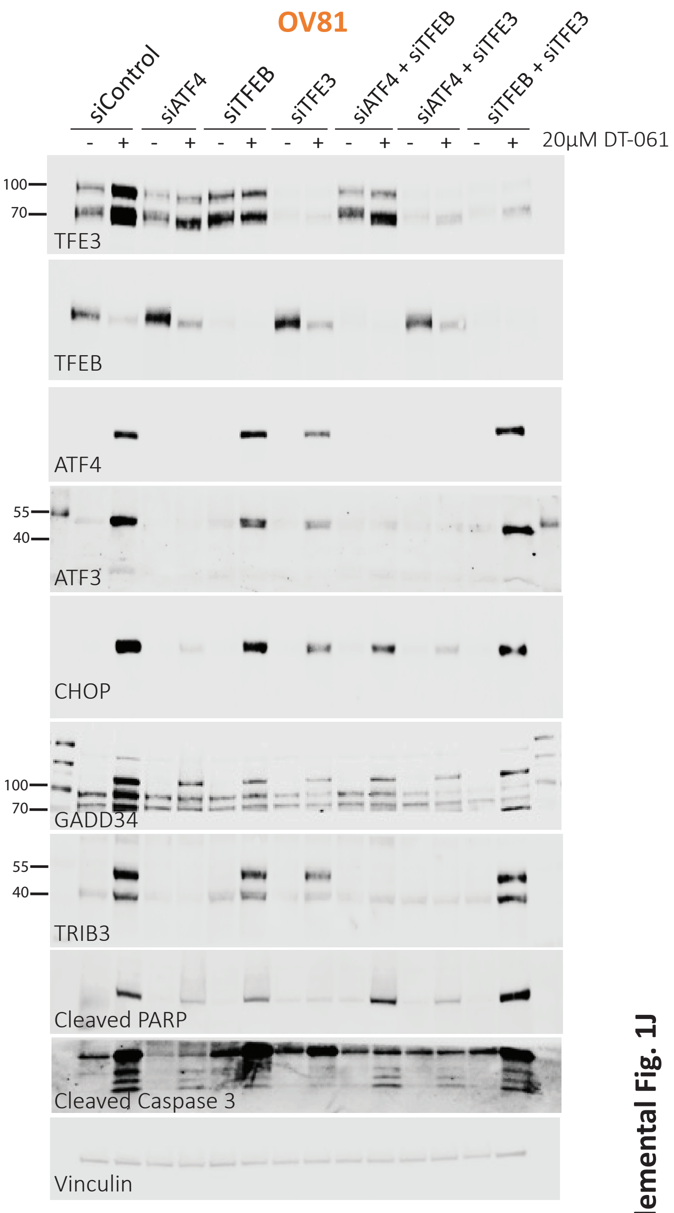

Fig. 3G

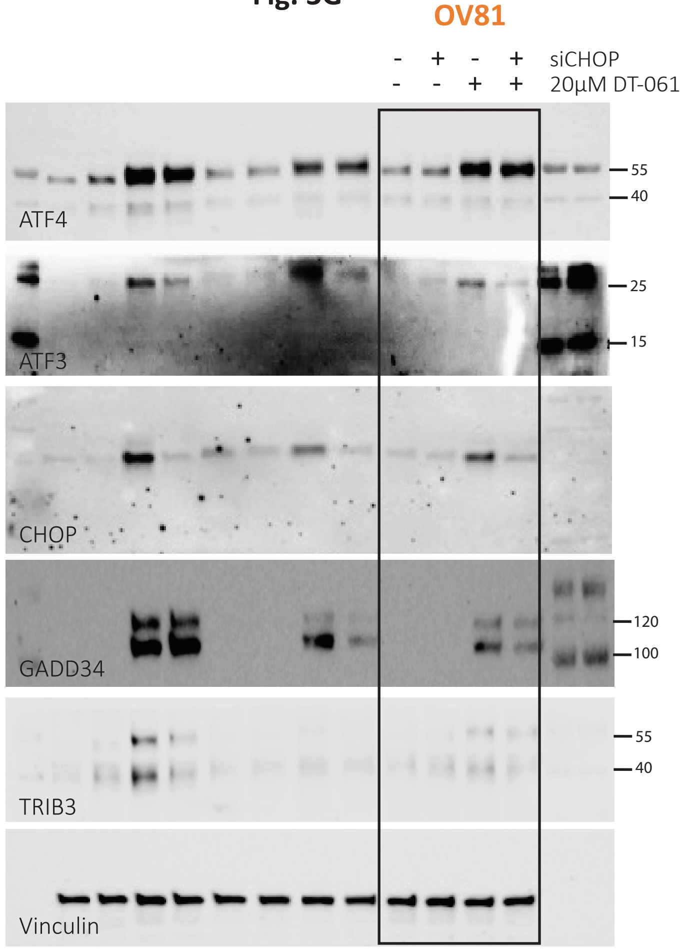

Supplemental Fig. 1J

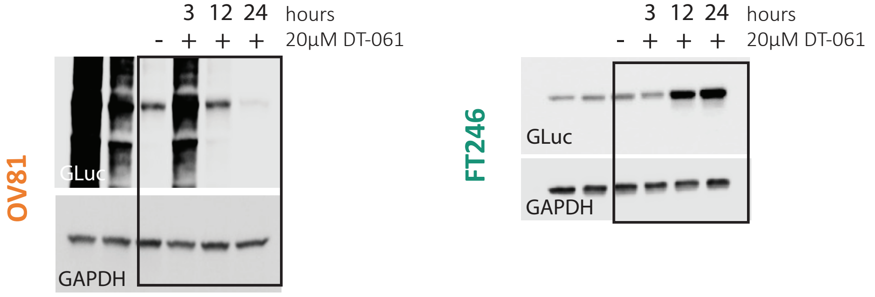

Fig. 3H

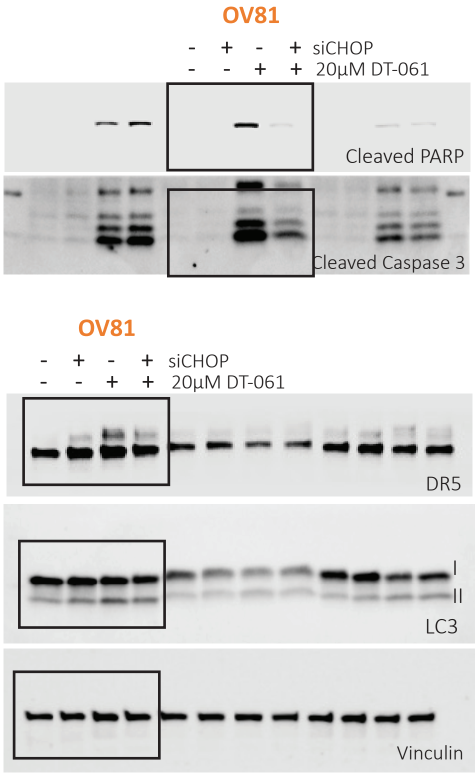

Supplemental Fig. 2H

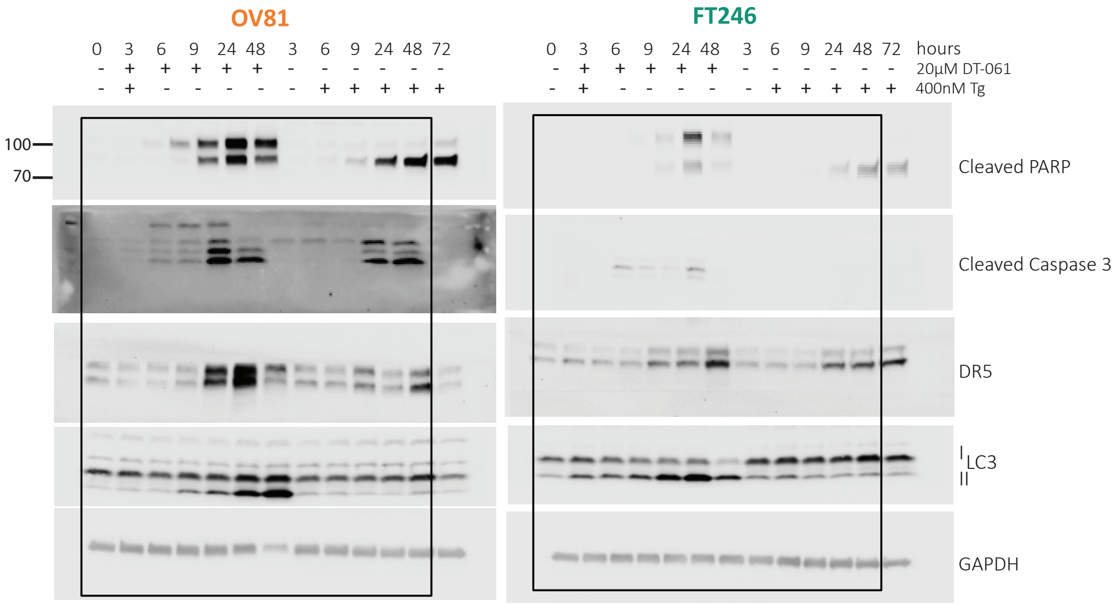

Supplemental Fig. 2O

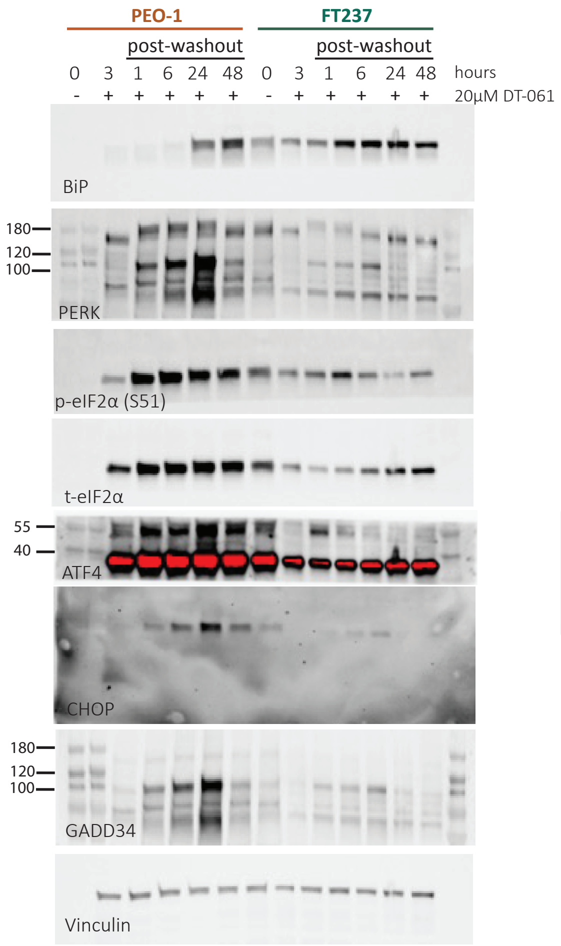

Supplemental Fig. 2L

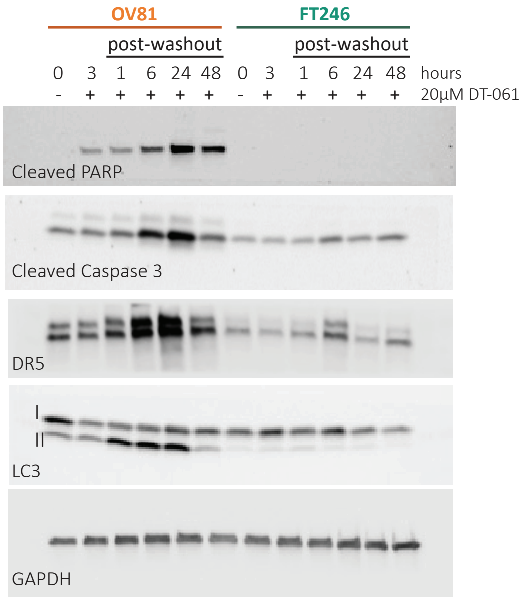

Supplemental Fig. 2P

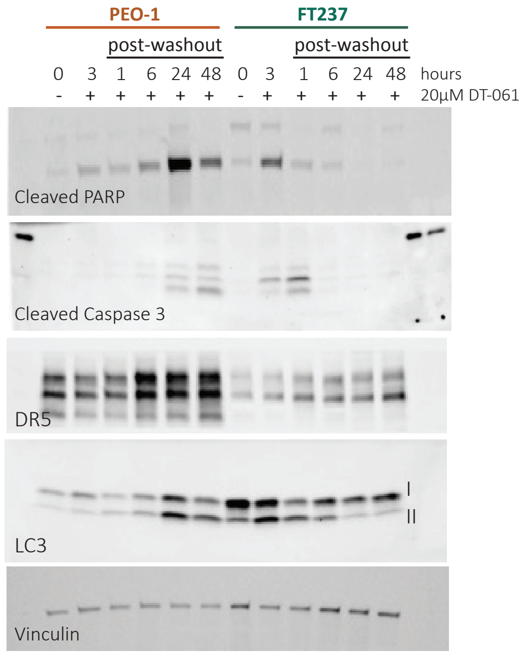

Supplemental Fig. 3A

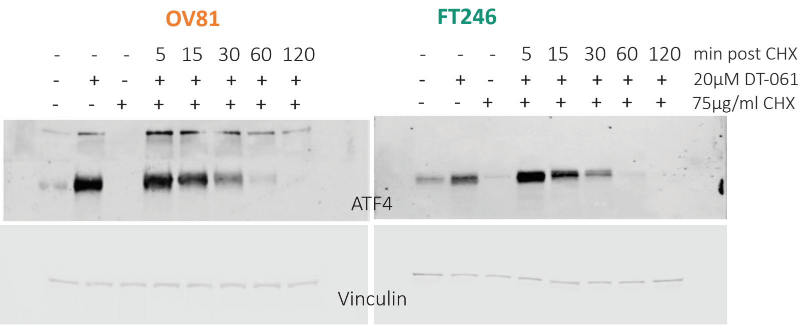

Supplemental Fig. 3B

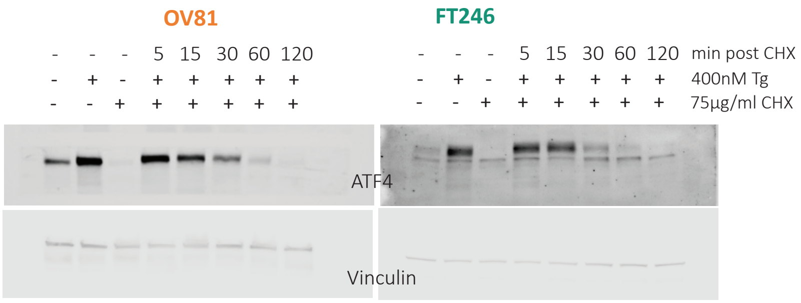

Supplemental Fig. 3D

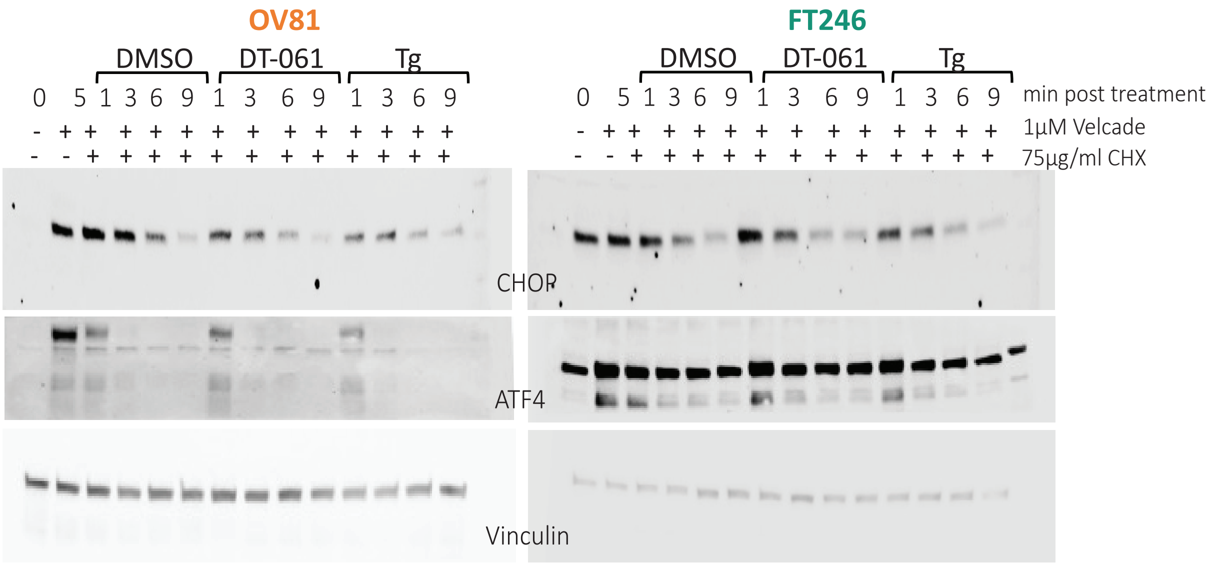

Supplement: Supplementary file 2 — Original Westerns [file 41418_2024_1378_MOESM2_ESM.pdf]
